# Peptide-Affinity Precipitation of Extracellular Vesicles and Cell-Free DNA Improves Sequencing Performance for the Detection of Pathogenic Mutations in Lung Cancer Patient Plasma

**DOI:** 10.3390/ijms21239083

**Published:** 2020-11-29

**Authors:** Catherine Taylor, Simi Chacko, Michelle Davey, Jacynthe Lacroix, Alexander MacPherson, Nicholas Finn, Gabriel Wajnberg, Anirban Ghosh, Nicolas Crapoulet, Stephen M. Lewis, Rodney J. Ouellette

**Affiliations:** 1 Atlantic Cancer Research Institute, Moncton, NB E1C 8X3, Canada; catherinet@canceratl.ca (C.T.); SimiC@canceratl.ca (S.C.); MichelleD@canceratl.ca (M.D.); Jacynthe.Lacroix@canceratl.ca (J.L.); AlexanderM@canceratl.ca (A.M.); GabrielW@canceratl.ca (G.W.); anirgho@gmail.com (A.G.); Nicolas.Crapoulet@vitalitenb.ca (N.C.); StephenL@canceratl.ca (S.M.L.); 2 Dr Léon-Richard Oncology Center, Moncton, NB E1C 8X3, Canada; Nicholas.Finn@vitalitenb.ca; 3 Department of Chemistry & Biochemistry, Université de Moncton, Moncton, NB E1A 3E9, Canada; 4 Beatrice Hunter Cancer Research Institute, Halifax, NS B3H 4R2, Canada

**Keywords:** biomarkers, lung cancer, exosomes, extracellular vesicles, liquid biopsy, genetic profiling, next generation sequencing

## Abstract

Liquid biopsy is a minimally-invasive diagnostic method that may improve access to molecular profiling for non-small cell lung cancer (NSCLC) patients. Although cell-free DNA (cf-DNA) isolation from plasma is the standard liquid biopsy method for detecting DNA mutations in cancer patients, the sensitivity can be highly variable. Vn96 is a peptide with an affinity for both extracellular vesicles (EVs) and circulating cf-DNA. In this study, we evaluated whether peptide-affinity (PA) precipitation of EVs and cf-DNA from NSCLC patient plasma improves the sensitivity of single nucleotide variants (SNVs) detection and compared observed SNVs with those reported in the matched tissue biopsy. NSCLC patient plasma was subjected to either PA precipitation or cell-free methods and total nucleic acid (TNA) was extracted; SNVs were then detected by next-generation sequencing (NGS). PA led to increased recovery of DNA as well as an improvement in NGS sequencing parameters when compared to cf-TNA. Reduced concordance with tissue was observed in PA-TNA (62%) compared to cf-TNA (81%), mainly due to identification of SNVs in PA-TNA that were not observed in tissue. *EGFR* mutations were detected in PA-TNA with 83% sensitivity and 100% specificity. In conclusion, PA-TNA may improve the detection limits of low-abundance alleles using NGS.

## 1. Introduction

Lung cancer is the leading cause of cancer deaths worldwide [1] and non-small cell lung cancer (NSCLC) accounts for approximately 90% of all lung cancer cases [2]. An improved understanding of driver mutations that fuel NSCLC malignancy has led to the development of targeted therapies that can prolong survival [3] and new targeted therapies, such as *PIK3CA* and *KRAS* inhibitors, are entering clinical trials every year [4]. Activating mutations in the epidermal growth factor receptor (*EGFR*) gene and rearrangements of the anaplastic lymphoma kinase (*ALK*) gene are the most common drivers in nonsquamous NSCLC for which approved drugs are available, but mutations in *BRAF* and *ERBB2*, as well as *MET* gene amplification and *MET* exon skipping and *ROS1* or *RET* gene fusions may also be potential targets for patient therapy [3,4]. Molecular profiling of biopsy tissue is therefore of paramount importance during lung cancer diagnosis in order to effectively determine a treatment strategy; however, recovering sufficient tissue for molecular analysis is a challenge, since many patients cannot undergo lung resection/biopsy due to contraindications [5], such as risk of a collapsed lung, or in other cases the tissue collected is either insufficient or of poor quality. Lack of molecular profiling is detrimental to patient care since it precludes eligibility for treatment with targeted therapies and enrollment into a clinical trial with biomarker inclusion criteria.

Liquid biopsy is a minimally-invasive diagnostic method that focuses on the detection of biomarkers that circulate in biofluids, such as blood, urine, saliva, cerebral spinal fluid, pleural effusion and others. Liquid biopsies can be performed using relatively small volumes of blood, urine, or other body fluids, and can facilitate a quicker turn-around time for molecular profiling results [6], as well as allow for longitudinal testing [7]. Circulating tumour DNA (ctDNA) isolated from plasma has been demonstrated to have high concordance with molecular alterations observed in the primary tumour [8,9]. Therefore it can be useful for selecting an appropriate targeted therapy, to assess the emergence of drug resistance mutations, and to monitor tumour burden during treatment; however, there is a great degree of variability in the amount of ctDNA present in plasma and the levels can be very low, representing 0.01% to 10% of the total cell-free DNA (cf-DNA) in the sample [10,11]. Therefore highly-sensitive analytical methods are required to detect rare mutant alleles in cf-DNA. Detecting DNA mutations in cf-DNA is especially useful when tumour biopsies are not available or practical, or when longitudinal sampling is required. Moreover, in many cases liquid biopsy may be the only available means of gaining useful molecular tumour information due to insufficient quantity or quality of DNA obtained from tumour tissue for next-generation sequencing (NGS) analysis of actionable mutations [12]. Liquid biopsy-based diagnostic assays exist for lung cancer and mainly focus on the detection of *EGFR* mutations in cf-DNA isolated from plasma. The cobas^®^ *EGFR* Mutation Test v2 is the first FDA-approved liquid biopsy test for lung cancer patients. Despite their many potential benefits, liquid biopsy approaches for tumour profiling in NSCLC still suffer from a lack of standardized techniques [13], limited coverage of relevant genes and pathogenic mutation hotspots, lack of sensitivity, and insufficient clinical validation [14].

Extracellular vesicles (EVs) are cell-derived nanoparticles, including exosomes, microvesicles, and apoptotic bodies, which are secreted from almost all cell types into the extracellular environment. EVs have been found in most body fluids, including blood, urine, milk, cerebrospinal fluid, semen, malignant effusions, and ascites, where they participate in both normal physiological and pathological processes. EVs, including exosomes (~30–150 nm) and microvesicles (~100–350 nm), mediate intercellular communication and contain cell-specific cargo such as growth factors, enzymes, receptors, cytokines, lipids, and coding and non-coding DNA and RNA molecules [15]. EVs can also be classified by size as small EVs (S-EVs) and large EVs (L-EVs) [16]. S-EVs generally include vesicles derived from the endosome-multivesicular body pathway, such as exosomes, and are < 200 nm in size. L-EVs are vesicles >200 nm in size that are derived from the plasma membrane and can include apoptotic bodies, microvesicles, and large oncosomes. L-EVs are becoming increasingly studied due to their distinct cargoes, functions, and relevance to cancer pathogenesis [17,18]. Increased numbers of EVs have been reported in blood and other biological fluids in response to cancers and other pathological conditions [19,20]. Circulating DNA has been reported to be mainly associated with EVs [21] and the use of EV capture in liquid biopsy analysis of ctDNA has been demonstrated to improve sensitivity over standard cf-DNA methods in human plasma [22,23,24] and pleural effusions [25]. Thus, the benefits of analyzing EVs in liquid biopsy applications is worthy of more study.

No single standardized method exists for the isolation of EVs for their use in diagnostic applications. The most commonly used methods for isolating EVs involve ultracentrifugation-based techniques that are time consuming and require specialized equipment not available at point-of-care sites, and thus can pose significant challenges for their adoption in clinical diagnostic labs. Other EV capture methods include immuno-affinity capture, which results in capture of subpopulations of EVs based on surface markers, polymer precipitation-based techniques, and size exclusion chromatography. Our laboratory has previously developed an EV isolation technology based on a synthetic peptide, Vn96, which has affinity for heat shock proteins [26] that are present on the surface of EVs [27]. This peptide-affinity (PA) EV isolation method uses low-speed centrifugation to precipitate peptide-EV complexes and has been adapted for use in a clinical diagnostic kit in Europe (SeleCTEV^TM^-DNA; Exosomics) [28,29]. One unique feature of Vn96 that other EV isolation methods do not share is the affinity of the peptide for directly capturing cf-DNA, even when it is not associated with extracellular vesicles [29,30], thereby maximizing recovery of nucleic acids from precious clinical samples.

In this study we tested the capability of Vn96 peptide-affinity (PA) capture of EVs and cf-DNA for genetic profiling of plasma from NSCLC patients and compared it to traditional cell-free methods. In order to maximize the detection of pathogenic mutations by liquid biopsy, we employed NGS and a lung cancer panel designed for cell-free total nucleic acid (cf-TNA) analysis and compared the relative performance of NGS using either cf-TNA or PA-captured total nucleic acid (PA-TNA) for the detection of mutant alleles in plasma. A small subset of NSCLC patients with tissue biopsies positive for *EGFR* mutations or deletions were also included in order to assess the sensitivity and specificity of PA-TNA for *EGFR* detection by NGS. A comparison of EDTA and Cell-Free DNA BCT (Streck) blood collection tubes on mutant allele detection by NGS was also made in this study in order to determine whether stabilization of white blood cells using Streck tubes to prevent genomic DNA contamination of the blood leads to improved detection of mutations. The concordance between both traditional cell-free and PA liquid biopsy methods was then analyzed and compared with mutations reported in matched tissue biopsies.

## 2. Results

### 2.1. Centrifugation of Plasma Does Not Significantly Impact DNA Yield, Size, or SNV Detection

**Figure 1:**
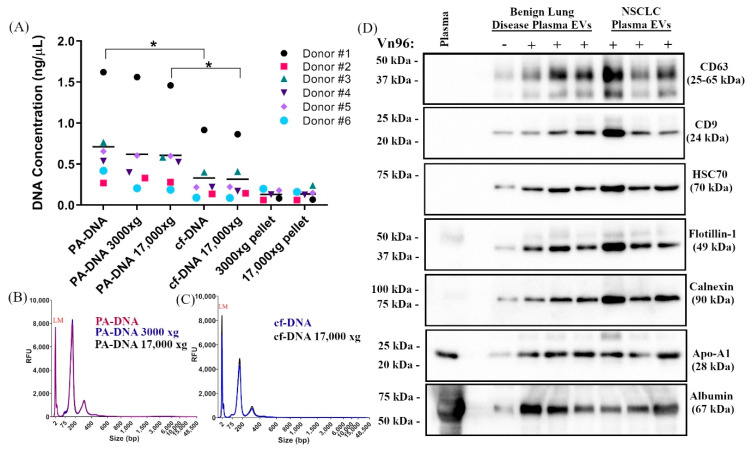
Effect of plasma pre-clearing on DNA recovery and detection of EV markers in PA precipitated material. (**A**) Plasma from six NSCLC donors was either not pre-cleared, or pre-cleared at 3000× *g* or 17,000× *g* for 15 min. The post-centrifugation pellet was retained, resuspended in nuclease-free water and DNA was extracted. Peptide affinity (PA) precipitation was performed on equivalent volumes of either non-precleared or pre-cleared plasma and DNA (PA-DNA) was isolated using the Plasma/Serum Cell-Free Circulating DNA Purification Mini Kit (Norgen Biotek). Cell-free DNA (cf-DNA) was obtained from equivalent volumes of plasma using the same DNA isolation kit for comparison (*n* = 6; * *p* < 0.05). A representative overlay of the DNA profiles of (**B**) PA-DNA or (**C**) cf-DNA from plasma (Donor #1) using either no pre-clearing or 3000× *g* or 17,000× *g* pre-clearing is shown. (**D**) A representative western blot (*n* = 3) of Vn96 PA precipitated material from 1 mL of plasma from donors with benign lung disease or NSCLC is shown. Canonical EV markers CD63, CD9, HSC70, and flotillin-1 were detected using specific antibodies. A vehicle control sample (without Vn96) was included as a negative control (−). In addition, calnexin, apolipoprotein A1 (Apo-A1), and albumin, which are common co-contaminants of EV isolations from plasma, were also detected using specific antibodies. Plasma protein lysate was included as a positive control for non-EV-associated plasma proteins.

Large EVs (L-EVs) have been reported to be the most abundant source of circulating tumour DNA that reflects the genomic make-up of the tumour of origin [31]. Many plasma pre-clearing protocols that are employed in liquid biopsy studies deplete this population of large vesicles in favour of enriching for small vesicles by subjecting plasma to centrifugation speeds of >10,000× *g* [32]. Since plasma processing protocols can greatly impact genomic DNA contamination and mutant allele detection [33], we first compared the effect of plasma pre-clearing centrifugation speed on the recovery of either cf-DNA or the DNA extracted from the combined capture of cf-DNA and EVs using peptide-affinity precipitation (PA-DNA). To determine whether pre-clearing plasma at either 3000× *g* or 17,000× *g* reduces the recovery of either cf-DNA or PA-DNA compared to no pre-clearing, we tested the effect of centrifugation speed on recovery of nucleic acids from plasma of six NSCLC patients (Figure 1A). Although centrifugation speeds of >6000× *g* have been reported to reduce recovery of circulating DNA from plasma [30,33], in this study no significant difference was observed in either cf-DNA or PA-DNA yield following centrifugation at 3000× *g* or 17,000× *g* compared to non-centrifuged plasma (Figure 1A, Supplementary Figure S2a). We did however find that PA-DNA recovery was significantly higher than cf-DNA from both non-precleared and pre-cleared plasma, consistent with the recovery of both EV-associated and circulating DNA by the Vn96 peptide (Figure 1A, Supplementary Figure S2a) which has been previously reported [29,30]. Both PA-DNA and cf-DNA yielded short fragments of DNA, with the majority being less than 200 bp in size, consistent with the size of circulating DNA reported by other groups [11,34]. This size profile was not significantly altered when plasma was pre-cleared by centrifugation at either 3000× *g* or 17,000× *g* (Figure 1B,C and Supplementary Figure S2).

Pre-clearing plasma is also thought to reduce contaminating genomic DNA, the presence of which may decrease the sensitivity of mutant allele detection [33]. In order to determine whether mutant allele detection can be improved by an additional plasma centrifugation step in this study, we used droplet digital PCR (ddPCR) to monitor the abundance of known mutant alleles in PA-DNA and cf-DNA that was extracted from the EDTA plasma of four NSCLC patients with or without an additional plasma centrifugation step at 3000× *g* or 17,000× *g* (Supplementary Figure S1b,c). Consistent with the fact that we did not observe significant amounts of high molecular weight (MW) DNA in either PA-DNA or cf-DNA regardless of centrifugation conditions, we also did not observe any differences in either the copies of mutant alleles detected or the mutant allele frequency following plasma pre-clearing by centrifugation. Based on the results of these experiments, we chose to omit a plasma pre-clearing step for the remainder of our studies in order to maintain the L-EV populations in our analyses.

### 2.2. PA Precipitation Enriches for Extracellular Vesicles

Western blot analysis was used to characterize EVs isolated using Vn96 peptide-affinity (PA) capture from EDTA plasma collected from patients with benign lung disease (*n* = 3) or NSCLC (*n* = 3) (Figure 1D). Canonical EV markers, including the tetraspanins CD63 and CD9, HSC70, and flotillin-1, were all observed in PA samples, but were very low in the absence of Vn96 peptide. The abundance of EV markers varied among individuals, but no differences were observed in EV marker abundance between patients with benign lung disease or NSCLC. PA-captured EVs also co-isolated non-EV markers, such as calnexin, the lipoprotein Apo-A1, and albumin. These co-contaminants suggest that protein aggregates containing Apo-A1 and albumin are likely trapped within the peptide-EV matrix and precipitate along with the EVs; however, lipoprotein bodies and albumin aggregates are not expected to interfere in the extraction or analysis of the cf-DNA and vesicle-associated DNA [29,30] that are recovered using peptide-affinity, since these contaminants are present at lower levels than are found in the plasma used for traditional cf-DNA methods.

### 2.3. PA Improves Recovery of Nucleic Acids from NSCLC Patient Plasma

A major aim of this study is the comparison between mutant allele detection in circulating nucleic acids versus PA-captured nucleic acids that have been extracted from NSCLC patient plasma and analyzed using NGS. Blood was collected from NSCLC patients in both EDTA tubes and cell-free DNA BCT^®^ tubes (Streck tubes) containing a preservative that prevents cell lysis and contamination of the blood with cellular DNA [33]. Plasma collected in Streck tubes was included in order to determine whether stabilization of white blood cells to prevent genomic DNA contamination of the blood leads to improved detection of mutations. Twenty patients with stage III/IV NSCLC for whom genetic profiling of tumour tissue was available were identified for this study. The clinicopathological characteristics of the NSCLC patients used in this study are shown in Table 1. A mutation or deletion in *KRAS*, *PIK3CA*, *TP53*, or *EGFR* was reported in the tumour biopsy of 80% of the patient cohort (Table 2).

**Figure 2:**
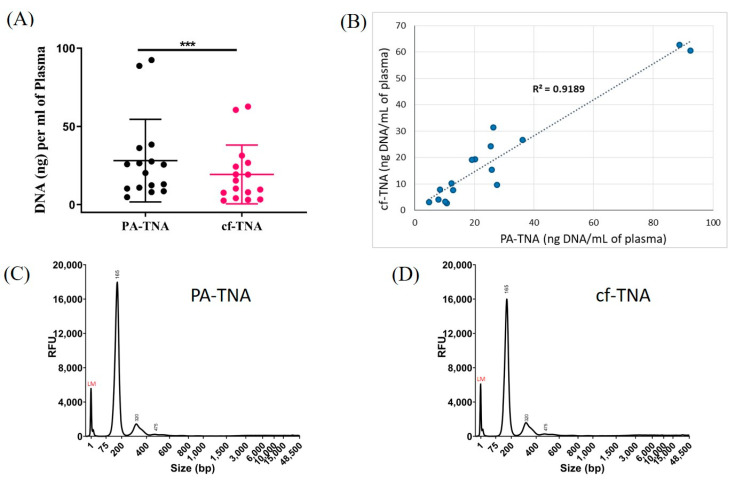
Peptide affinity precipitation improves recovery of nucleic acids compared to cell-free method. Total nucleic acid (TNA) was either extracted directly (cf-TNA) from non-pre-cleared EDTA plasma from sixteen NSCLC donors or from PA-precipitated material (PA-TNA) from an equivalent volume of plasma. TNA was isolated using the MagMax Cell-Free Total Nucleic Isolation Kit and DNA was quantified using the High Sensitivity DNA assay on a Qubit fluorometer. (**A**) The recovery of DNA per mL of plasma and (**B**) the correlation between DNA recovery in PA-TNA or cf-TNA isolation methods is shown (*n* = 16; *** *p* < 0.001). A comparison of the DNA profiles obtained from assaying PA-TNA (**C**) and cf-TNA (**D**) on a Fragment Analyzer are shown.

Vn96 peptide-affinity capture is a unique EV isolation method for mutation analysis in that it is able to capture not only EV-associated DNA but also circulating free DNA [29,30]. EDTA plasma from 16 matched samples was used to isolate total nucleic acid using either the PA method or a conventional cell-free method that employed a semi-automated bead-based total nucleic extraction method recommended for detection of both DNA mutations and RNA gene fusions with the Oncomine™ Lung Cell-Free Total Nucleic Acid Panel for NGS. The panel covers single nucleotide variants (SNVs) in ten genes including *ALK*, *BRAF*, *EGFR*, *ERBB2*, *KRAS*, *MAP2K1*, *MET*, *NRAS*, *PIK3CA*, and *TP53* (168 hotspots) as well as copy number variations (CNVs) in *MET* and gene fusions in *ALK*, *RET*, and *ROS1*. PA precipitation has been reported to yield more DNA from plasma than cf-DNA isolation and other EV isolation methods [29]. Consistent with previous reports regarding its ability to capture both cf-DNA and EVs [29,30] in this study the PA method resulted in a higher recovery of DNA from equivalent volumes of matched plasma than the conventional cell-free method (Figure 2A). Although PA increased the recovery of DNA from patient plasma, the recovery of DNA between the PA and cell-free methods from individual patients was closely correlated (Figure 2B). The size profiles of DNA were strikingly similar for PA-TNA and cf-TNA liquid biopsy methods, with the major peak at ~ 160–170 bp and with very little high MW DNA observed in either sample type (Figure 2C,D; Supplementary Figure S3).

### 2.4. PA Improves NGS Performance

**Figure 3:**
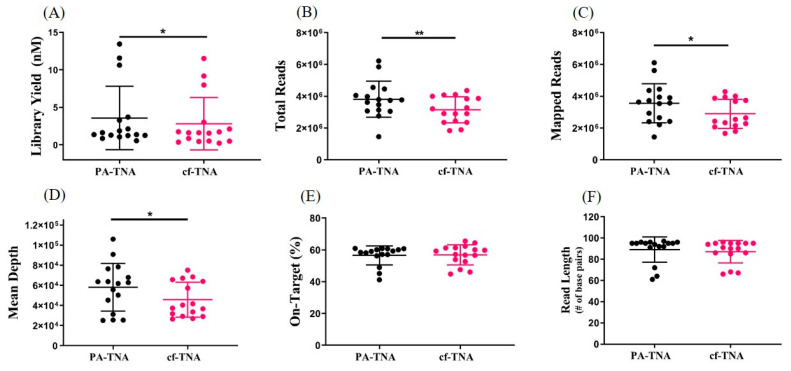
PA improves performance of next generation sequencing compared to cf-TNA. PA-TNA or cf-TNA extracted from EDTA plasma from NSCLC patients was sequenced by Next Generation Sequencing using an Oncomine Cell-Free Total Nucleic Acid panel. Up to 50 ng of PA-TNA or cf-TNA isolated from up to 4 mL of EDTA plasma was used to generate barcoded libraries. The libraries were quantified by Tapestation (**A**), multiplexed and amplified on an Ion Chef instrument and sequenced on an Ion Genestudio S5 sequencer. Comparisons of different NGS parameters including total reads (**B**) mapped reads (**C**) mean sequencing depth (**D**) percent of on-target reads (**E**) and mean read length (**F**) are shown. (*n* = 16; * *p* < 0.05, ** *p* < 0.01).

Up to 50 ng of PA-TNA or cf-TNA isolated from paired EDTA plasma samples was used to prepare libraries using target specific amplification. Amplicons were then purified, barcoded, and the libraries were quantified using a Taqman library quantification assay. Each individual library was diluted, pooled and normalized to 50 pM prior to templating and sequencing on an Ion S5 Gene Studio sequencer. Libraries constructed using PA-TNA improved library yield compared to cf-TNA (Figure 3), which may be related to increased DNA recovery and consequently higher input of PA-TNA into the library preparation compared to cf-TNA; however, only a weak correlation (R^2^ = 0.56) was observed between DNA input and library yield (Supplementary Figure S6). Therefore, other factors, such as the absence of inhibitors of amplification, may also have contributed to improved library yield for PA-TNA compared to cf-TNA. Despite the fact that library quantities were normalized among all samples prior to sequencing, increased numbers of total and mapped reads and increased sequencing coverage were observed when PA-TNA was used as a template for NGS rather than cf-TNA (Figure 3); however, no differences in the percent of on-target reads or read length were observed (Figure 3). Therefore, the use of PA precipitation for liquid biopsy not only increases the recovery of DNA from plasma, but also improves NGS performance compared to conventional cell-free methods.

### 2.5. PA Improves Mutant Allele Detection

**Figure 4:**
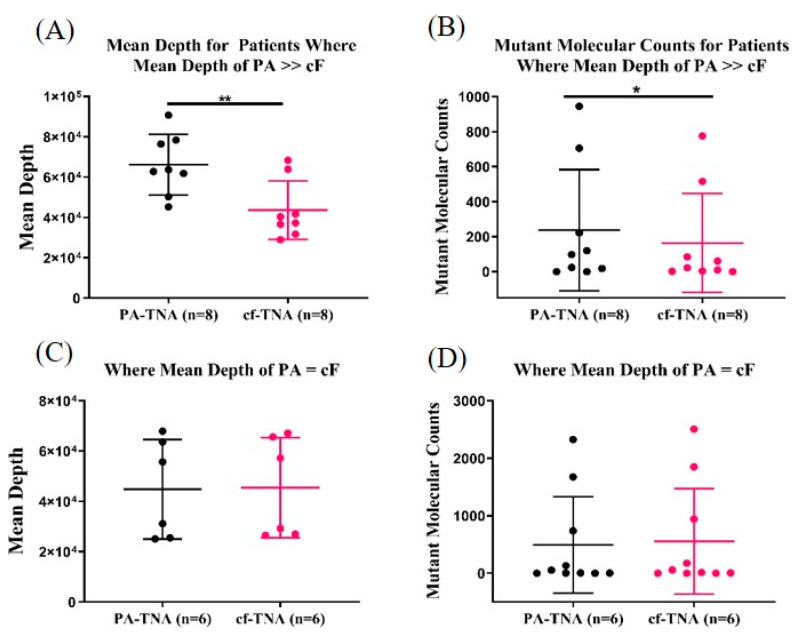
Improved NGS performance correlates to increased mutant molecular count in PA. Mutant allele detection was compared in PA-TNA or cf-TNA extracted from EDTA plasma from matched NSCLC patients and sequenced by Next Generation Sequencing using an Oncomine Cell-Free Total Nucleic Acid panel. Samples were divided into two groups based on relative mean sequencing depth between paired PA-TNA and cf-TNA samples. The first group (*n* = 8; * *p* < 0.05, ** *p* < 0.01) contained patient samples in which mean depth of PA-TNA was >10% higher than the paired cf-TNA sample (**A**) and the mutant molecular count for mutations detected within this group were compared (**B**). The second group (*n* = 6) contained patient samples in which mean depth of PA-TNA was roughly equivalent (<10% difference) to the paired cf-TNA sample (**C**) and the mutant molecular count for mutations detected within this group were compared (**D**).

PA precipitation has been reported to improve sensitivity of *BRAF*-V600E detection in melanoma patient plasma [29] and to improve detection of low-frequency mutations in prostate cancer patients [28] by ddPCR analysis. In order to determine whether the increased sequencing depth observed with PA-TNA samples is correlated with improved SNV detection, we compared the number of counts of mutant alleles in patient samples (8/15) where the mean sequencing depth of PA-TNA was greater than 10% higher than paired cf-TNA (Figure 4A). The same comparison was made in patient samples (6/15) where the sequencing depth was roughly equivalent between paired PA-TNA and cf-TNA samples (<10% difference in sequencing depth; Figure 4C); only one patient sample had a sequencing depth for PA-TNA that was less than that of paired cf-TNA (data not shown). When PA-TNA samples with improved sequencing depth were grouped together, the average of mean depth of the PA-TNA samples was 66,180 compared to 43,627 for cf-TNA samples (*n* = 8; *p* = 0.008). When the read counts of the mutant alleles detected by NGS in this group of samples were compared, we observed a statistically significant increase in the number of mutant molecular counts in PA-TNA samples compared to cf-TNA (Figure 4B); however, when samples with equivalent sequencing depth were grouped together (Figure 4C; 44,823 for PA-TNA and 45,463 for cf-TNA [*n* = 6, *p* = 0.3]), no difference in mutant molecular counts was observed (Figure 4D). A similar comparison was made between cf-TNA isolated from either EDTA or Streck tubes and a similar trend of increased mutant molecular counts in samples with higher mean depth was also observed (Supplementary Figure S7). These data demonstrate that improved sequencing depth by using PA-TNA as an NGS template rather than cf-TNA is correlated with improved detection of mutant alleles in these samples.

### 2.6. Concordance Observed between Liquid and Tissue Biopsies

**Figure 5:**
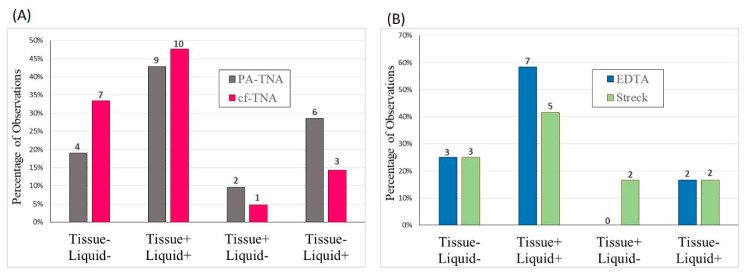
Concordance of mutations detected in tissue biopsy and liquid biopsies. (**A**) Mutant allele detection by NGS was compared between PA-TNA or cf-TNA extracted from EDTA plasma from matched NSCLC patients and compared to mutations reported in the tissue biopsy. (**B**) Mutant allele detection by NGS was compared between cf-TNA extracted from EDTA or Streck plasma from matched NSCLC patients and compared to mutations reported in the tissue biopsy.

Next, we examined the concordance among mutations identified by NGS in the tumour biopsy material and those observed for matched plasma subjected to PA precipitation (PA-TNA) or standard cell-free methods (cf-TNA). Since different NGS panels were used for tissue biopsy and plasma liquid biopsy sequencing, only variants covered by both panels were considered in the analysis (Figure 5). Overall, good concordance was observed among tissue and both liquid biopsy methods, with 81% concordance (17/21 total mutant alleles) for cf-TNA and 62% concordance (13/21 total mutant alleles) for PA-TNA, with PA-TNA missing one mutant allele that was found in both tissue and cf-TNA. The decrease in the concordance that was observed for PA-TNA compared to cf-TNA was mainly due a decrease in concordance of tissue samples without mutations (Tissue- Liquid-), as well as the detection of genetic variants in PA-TNA samples that were not detected in the tissue (Tissue- Liquid+). In three of these cases, genetic alterations detected in PA-TNA were not detected in either tissue or in cf-TNA, leading to higher tissue concordance rates for cf-TNA (Figure 5). Therefore, although cf-TNA has better concordance with tissue when no mutations are present, in three instances PA-TNA detected variants that were not detected in the tissue biopsy. Where possible, ddPCR was used to confirm the SNVs detected for liquid biopsy samples by NGS (Supplementary Figure S8). Two mutations that were not detected in the tissue biopsy, but were detected in PA-TNA and cf-TNA, by NGS were also confirmed in both PA-TNA and cf-TNA by ddPCR. Four additional variants detected in PA-TNA and cf-TNA by NGS were also confirmed by ddPCR (Supplementary Figure S8). In all cases where ddPCR was used to confirm mutations detected using NGS, the mutant allele frequency (MAF) observed was very similar using both methods. Therefore, both cf-TNA and PA-TNA liquid biopsy methods offer good concordance with SNVs detected in the tissue biopsy; however, mutations that were not observed in the tissue biopsy were more likely to be found in PA-TNA than cf-TNA (Supplementary Figure S9), likely due to increased sequencing depth. Although the sample number was small, concordance between EDTA cf-TNA and tissue was better than for Streck cf-TNA (Figure 5). EDTA cf-TNA had 83% (10/12) concordance with tissue while Streck cf-TNA had 67% concordance (8/12), suggesting that EDTA blood collection tubes may be preferable for NGS sampling in cases where blood will not be stored for long in the collection tube.

### 2.7. High Specificity of EGFR Mutant Allele Detection Observed Using PA

*EGFR* mutations or deletions are one of the most common driver mutations found in NSCLC adenocarcinoma and reliable identification of alterations in *EGFR* in NSCLC patients is critical to correctly identify patients that will benefit from treatment with tyrosine kinase inhibitors. Six NSCLC patients in our study had tissue biopsies with confirmed *EGFR* mutations or deletions. We were able to detect the same *EGFR* alteration in PA-TNA that had been reported in the matched tissue in 5 of 6 patients (83% sensitivity; Table 3 and Table 4). Furthermore, *EGFR* alterations were detected with 100% specificity in our study using PA-TNA (Table 4). Therefore, PA-TNA liquid biopsy can improve NGS performance resulting in improved mutant allele detection, particularly of low frequency variants.

## 3. Discussion

With the advent of personalized medicine and the rapid increase in the development of targeted therapies for cancer treatment, establishing the molecular profile of an individual’s cancer has become of paramount importance in order to select therapies that are most likely to be effective. Testing for oncogenic activation of tyrosine kinases, such as those mediated by *EGFR* mutations and *ALK* rearrangements, has become standard practice during diagnostic evaluation of NSCLC adenocarcinoma patients and has led to wide adoption of targeted therapies and greatly improved outcomes for patients with these types of genetic modifications [35]. Although sequencing of tissue biopsies to obtain a tumour molecular profile leads to improved disease management, difficulties in obtaining sufficient tumour tissue for sequencing, long turnaround times for biopsy procedures and tissue biomarker testing [35], as well as contraindications that can prevent patients from undergoing tissue biopsy, has been reported to prevent 40–50% of patients [35] from benefiting from genetic tumour profiling.

The discovery that tumours shed ctDNA into the circulatory system, which carries the same somatic mutations that are found in the tumour [31], has led to the rise in popularity of minimally-invasive liquid biopsy diagnostics. The majority of extracellular DNA has been reported to be associated with EVs, either encapsulated within the vesicle or on the surface membrane [36], and represents an unbiased coverage of the entire genome [36]. L-EVs in particular are shed by cancer cells and are much more abundant in plasma from cancer patients compared to healthy donors and have been reported to contain histone-associated dsDNA [31]. The release of genomic DNA into blood due to lysis of white blood cells (WBC) post-blood draw is a known complication of liquid biopsies [37] and can lower sensitivity of mutation detection [33]. Pre-clearing plasma prior to EV capture is therefore common practice in order to remove cell debris and contaminating genomic DNA released from lysed WBCs. Many studies focused on the use of EVs for liquid biopsy have used protocols to pre-clear plasma that include centrifugation speeds of 10,000× *g* or greater prior to EV capture. These centrifugation speeds are known to deplete L-EVs, including microvesicles and oncosomes [16,31], which have been reported to encapsulate the majority of extracellular DNA in plasma [31] and mirror the genetic profile of the tumour in cancer patients better than S-EVs [31]. Although we did not observe a significant reduction in DNA yield or changes to the DNA molecular weight profile after centrifugation of 3000× *g* or 17,000× *g*, the plasma used in our study was processed within 30 min of blood collection to minimize platelet activation [38] and WBC lysis [37]. In cases where this cannot be achieved, pre-clearing at 3000× *g* or use of Streck blood collection tubes may be beneficial to reduce contaminating genomic DNA [39] without significantly depleting the L-EV population. In one patient, we did observe high MW DNA in PA-TNA but not in cf-TNA (Supplementary Figure S3); however, since this patient did not have a tumour mutation that was covered by the sequencing panel we cannot determine if this high MW DNA was released from the tumour encapsulated in L-EVs or oncosomes. Vagner et al. demonstrated that L-EVs contain chromosomal high MW DNA that is derived from tumour cells [31], so this is one possible source of the contaminating high MW DNA. It is also possible that the DNA originates from lymphocytes that were lysed and released their genomic DNA into circulation prior to plasma preparation, although one might reasonably expect lymphocyte-derived DNA to also be present in cf-DNA if this were the case.

To compare the effect of the blood collection tube on the recovery of cf-TNA, cf-TNA was extracted from seven paired NSCLC blood samples collected in either EDTA or Streck tubes. We did not observe any differences in DNA yield between EDTA and Streck tubes (Supplementary Figure S4), which is consistent with other reports where increases in DNA yield due to lymphocyte lysis were only observed in EDTA plasma after prolonged incubation of blood in EDTA tubes [39]. Likewise, no significant differences were observed in library yields, read number or sequencing depth between cf-TNA extracted from EDTA or Streck tubes (Supplementary Figure S5). Our observation that there are no significant differences in mutant allele detection between EDTA and Streck blood collection tubes also suggests the lack of lymphocyte genomic DNA interfering with mutant allele detection in our study.

Many factors can affect the size and characteristics of circulating EVs, including method of blood collection, sex, age, fasting/non-fasting, time of day, recent exercise, both infectious and non-infectious disease, as well as other conditions such as pregnancy [16]. These factors may have contributed to the biological variability of precipitated EV protein markers observed in Figure 1D. Non-EV lipid structures, such as low and high density lipoprotein particles, are very abundant in plasma, share similar size and density with EVs, and are a common contaminant of EV isolations [16]. As is the case with many EV isolation methods, co-isolation of high-density lipoprotein Apo-A1 and albumin with PA-isolated EVs was observed in this study; however, a recent study observed less co-isolation of serum albumin and lipoproteins with PA-isolated EVs compared to a centrifugation-based EV isolation method and size-exclusion chromatography, respectively [40].

Extracellular vesicle analysis in liquid biopsy has several advantages over cf-DNA analysis, including enhanced stability of their cargo due encapsulation within the lipid bilayer of the vesicle and EV cargo may better reflect the parent cell than cf-DNA because EVs are secreted from living cells rather than shed from dying cells like cf-DNA [41]. Although controversial due to reports that double-stranded DNA and histones are absent from S-EVs [42], other studies have demonstrated the presence of DNA in exosomes [43] and L-EVs [31] and indeed have reported that most extracellular DNA in plasma in localized in EVs [21,31]. High MW DNA that accurately reflects tumour genetic diversity has been described in L-EVs [31]. A number of studies have reported that EV DNA improves mutation detection sensitivity and specificity compared to cf-DNA [25,29,44] and that use of EVs can improve detection of circulating biomarkers [45]. Although most studies comparing EVs and cf-DNA monitored the presence of mutations in a single gene (e.g., *EGFR* or *BRAF*) by PCR, more comprehensive genetic testing to identify less common targetable alterations in *BRAF*, *ROS1*, *RET*, *NTRK*, *MET*, and *ERBB2* could also improve outcomes for a subset of NSCLC patients [35].

As an increasing number of gene modifications that are important in the pathophysiology of lung cancer are discovered, and with an ever-increasing arsenal of targeted therapies in development, we have opted for a broader comparison of liquid biopsy to tissue biopsy by employing a panel-based NGS method that covers more than 150 hotspots in eight different genes important in lung cancer. As expected, due to its affinity for both cf-DNA and EVs, PA-captured material from plasma contains higher concentrations of nucleic acid than the cell-free method. Alborelli et al. reported that the amount of cf-DNA input has a direct impact on sequencing performance [46]. We also found that PA-TNA, where higher DNA input into the library preparation was possible due to better DNA recover compared to cf-TNA, improves the yield of NGS libraries as well as sequencing performance. The correlation of DNA input to library yield was weak, which suggests other factors may be involved. One possibility is that cf-TNA may contain inhibitors that can impair NGS library construction that are absent, or present at lower levels, in PA-TNA. We also observed an improvement in the number of mapped reads and sequencing depth with PA-TNA, although no differences in read length or percent-on target reads were observed. Since equivalent amounts of library were inputted for all sequencing reactions, these results suggest that the quality of the NGS libraries used for sequencing are generally better when PA-TNA is used as the template rather than cf-TNA. Although we did not observe any differences in the DNA length profile between PA-TNA and cf-TNA, one possible explanation is that PA-TNA also contains DNA that is protected from degradation by encapsulation within the lipid bilayer of the vesicle.

Previous studies have reported *EGFR* mutation status concordance between liquid and tissue biopsies in NSCLC to range from 80% to 94% [47,48,49]. Although very few multi-gene analysis studies have been performed in NSCLC, meta-analysis of studies comparing the concordance rate of liquid and tissue biopsies from pancreatic cancer patients using NGS multi-gene panels found a concordance rate of only 31.9% [50]. In this study we observed 81% concordance for cf-TNA and 62% concordance for PA-TNA compared to matched tissue biopsies. The lower concordance with tissue observed for the PA-TNA group was mainly due to an increase in observed genetic alterations that were not observed in either tissue or cf-TNA. Pathogenic mutations were detected in plasma PA-TNA from 6 patients that were not detected in the matching tissue biopsy. The ability of PA to more sensitively detect low frequency mutations and the emergence of tumour subclones than cf-DNA has been previously reported [28,29]. Since ct-DNA is considered to be a good representation of the tumour genetic diversity and therefore may better represent a variety of tumour subclones than DNA isolated from a single site in a tumour biopsy [6,12,51], it is possible that the identification of these mutations is the result of tumour heterogeneity that is better represented in the blood; however, we cannot discount the possibility that one or more of these mutations could be somatic mutations present in lymphocyte DNA that circulates in the plasma [52]. Although the genetic profiles resulting from PA and cell-free methods generally matched each other, PA-TNA sequencing detected genetic alterations in three patients that were not observed with cf-TNA, likely due to increased sensitivity of mutant allele detection resulting from the higher number of sequencing reads observed in PA-TNA in samples from those three patients. Due to the presence of circulating tumour DNA in cf-DNA, liquid biopsy methods have been proposed to provide a better representation of tumour heterogeneity than solid biopsies from a single lesion [53].

*EGFR* mutations detected in plasma predict response to *EGFR* tyrosine kinase inhibitor treatment at a similar efficacy as *EGFR* variants detected in tumour tissue [54]; moreover, the sensitivity (66–90%) and high specificity (>95%) of *EGFR* mutation detection from plasma cf-DNA proves it is a viable surrogate for *EGFR* mutation detection in advanced NSCLC [47,48]. Isolation of plasma EVs has been reported to further increase sensitivity in comparison to cell-free methods [22,23,44]. Although the sample size was very small we observed very good concordance (83%) of *EGFR*+ patients with 100% specificity, indicating that PA-TNA can reliably capture *EGFR* mutations in circulating and/or EV-DNA. Indeed, for one patient in our study, their *EGFR*+ status was first determined using PA-TNA and subsequently confirmed in tissue during a repeat biopsy, further highlighting the need to adopt liquid biopsies in the clinical setting.

Interest in using EV cargo to identify novel biomarkers related to cancer diagnosis, prognosis, and response to therapies has spiked in recent years [45,55]. The EV lipid bilayer not only provides protection from degradation for extracellular DNA [21], but also protects a wide variety of RNA and proteins that reflect the vesicle’s cellular origin [45,55,56]. The use of peptide-affinity precipitation for liquid biopsy has an additional advantage over traditional cell-free methods in that it facilitates the enrichment of multiple analytes from plasma and other biofluids that can be mined for biomarker discovery. Simultaneous extraction of DNA, RNA and protein using PA-isolated EVs from a single plasma sample [30] not only maximizes the information that can be gathered from valuable, and limited, clinical samples, but also enables a multi-analyte approach to identify cancer signatures that better reflect the complexity of the disease. EV membrane-associated proteins, including NY-ESO-1, *EGFR* and *EpCAM*, have been found to have prognostic value in cancer patients [57]. EVs contain a wide range of RNA types with a reported prevalence of non-coding RNA, including microRNA (miRNA), as well as messenger RNA. Many studies have reported on the potential use of EV-encapsulated miRNAs as biomarkers in cancer diagnosis and prognosis [56]. Therefore, use of EVs in liquid biopsy applications facilitates enrichment of novel biomarkers and may lead to new diagnostic tests for cancer patients.

Widespread clinical adoption of ctDNA analysis as an integral part of cancer management is foreseeable if issues related to specificity and sensitivity of these methods can be addressed. Maximizing the sensitivity of liquid biopsy diagnostics is of paramount importance, since SNVs are often present at very low frequencies in the circulation. PA-mediated ct-DNA and EV capture may be of benefit in this context due to improved NGS performance and the resulting increase in sensitivity of mutant allele detection. Clinical adoption of liquid biopsies to improve the accessibility of molecular profiling for NSCLC patients could improve clinical outcomes by ensuring patients can receive appropriate targeted therapies, even when a tissue biopsy is not available or possible. This study demonstrates an NGS-based liquid biopsy approach that allows a more comprehensive assessment of an individual patient’s tumour genetic profile and could also improve eligibility for clinical trials with biomarker inclusion criteria.

## 4. Materials and Methods

### 4.1. Patient Samples

Blood from patients with benign lung disease or stage III/IV NSCLC was collected in both EDTA and cell-free DNA BCT^®^ (Streck, Omaha, NE, USA) blood collection tubes. Approval for this study was obtained from the Research Ethics Board of Vitalité Health Network (REB number: CER-2017-30; approved Nov 9 2017) and written informed consent was obtained from the patients. The study was performed in accordance with the Canadian Research Tri-Council policy on ethical conduct for research involving humans (https://ethics.gc.ca/eng/policy-politique_tcps2-eptc2_2018.html).

### 4.2. Plasma Processing

Blood was processed within 30 min of collection by centrifugation in a benchtop centrifuge at 1500× *g* for 15 min. The plasma fraction was collected, aliquoted, and stored at −80 °C. Plasma was thawed at room temperature and then either used directly (no pre-clearing), or was pre-cleared by centrifugation at either 3000× *g* or 17,000× *g* for 15 min. The plasma supernatant was transferred to a new tube prior to use.

### 4.3. Peptide-Affinity EV and cf-DNA Precipitation

The Vn96 peptide (PSQGKGRGLSLSRFSWGALTLGEFLKL) was previously developed in our laboratory [26]. The peptide was prepared at 2.5 mg/mL in peptide resuspension buffer [0.625× PBS + 0.05% Pro-Clin300 (cat # 48912-U, Sigma,St.Louis, MO, USA)]. Precipitation of EVs and cf-DNA from EDTA plasma was performed by diluting 1 mL of plasma in 1 mL of 1× PBS and then adding 0.25% (*v*/*v*) protease inhibitor cocktail (cat# 539134, Cedarlane, Burlington, ON, Canada)) and 0.1 mg/mL of Vn96 peptide. The Vn96/plasma solution was rotated end-over-end for 1 h at room temperature and then centrifuged at 17,000× *g* for 15 min to pellet the peptide/EV/cf-DNA complexes. The pellet was washed 3 times with 1× PBS followed with centrifugation at 17,000× *g* for 10 min between washes. To control for non-specific precipitation, a vehicle control consisting of an equivalent volume of peptide resuspension buffer was included in western blotting experiments.

### 4.4. Western Blotting

Peptide-affinity precipitated material was resuspended in 60 µL of protein lysis buffer (2% SDS, 150 mM Tris-HCl pH 6.8, 0.5% protease inhibitor cocktail III). The samples were heated at 70 °C for 15 min and then 15 µL of 4× Laemmli sample buffer (Bio-Rad, Hercules, CA, USA) was added and the samples were heated again at 70 °C for 15 min. The lysates were vortexed and then an aliquot was removed and directly loaded onto a 12% SDS-PAGE gel while the remainder was reduced with Bond-Breaker™ TCEP solution (Fisher Scientific, Waltham, MA, USA) and heated at 95 °C for 5 min. Protein lysates were separated by SDS-PAGE and western blotting for canonical EV protein markers and common plasma contaminants was performed. Non-reduced protein lysate was used for detection of the glycosylated CD63 protein. Antibodies against CD63 (MX-49), CD9 (ALB 6), HSC70 (B-6), Apo-A1 (B-10) were obtained from Santa Cruz BioTechnology(Dallas, TX, USA). The antibodies against flotillin-1 (D2V7J) and albumin (cat# 4929) were obtained from New England Biolabs (Ipswich, MA, USA), while the calnexin antibody (cat# ab22595) was obtained from Abcam (Cambridge, UK)

### 4.5. Nucleic Acid Isolation

*DNA Isolation:* DNA was isolated directly from up to 0.5 mL of plasma (cf-DNA) using the Plasma/Serum Cell-Free Circulating DNA Purification Kit (Norgen Biotek, Thorold, ON, Canada) according to the manufacturer’s instructions. DNA was extracted from peptide-affinity purified material (PA-DNA) using the same kit after resuspending the PA-precipitated pellet containing EVs and cf-DNA in 0.5 mL of PBS prior to extraction. *TNA Isolation:* total nucleic acid was isolated directly from 2 mL of plasma (cf-TNA) or from peptide-affinity purified Evs and cf-DNA precipitated from plasma (PA-TNA) and resuspended in 2 mL of PBS. Total nucleic acid was extracted using the MagMAX Cell-Free Total Nucleic Acid Isolation Kit (MagMax cfTNA; cat # A36716, ThermoFisher Scientific, Waltham, MA, USA) according to the manufacturer’s instructions using a KingFisher™ Duo Prime Magnetic Particle Processor with 6- and 12-well magnetic heads. The eluted TNA was quantified on Qubit™ 2.0 Fluorometer (Invitrogen, Carlsbad, CA, USA) using the Qubit™ dsDNA HS Assay (Invitrogen). In the event that insufficient DNA/RNA was recovered from 2 mL of plasma, a second extraction from 2 mL of plasma was performed and pooled with the nucleic acids from the first extraction.

### 4.6. Fragment Analyzer

To assess cf-DNA and PA-DNA size distribution and the presence of genomic DNA, samples were separated by electrophoresis on an Agilent 5200 Fragment Analyzer System using the high sensitivity (HS) Genomic DNA 50 kb kit (DNF-468, Agilent Technologies Inc., Palo Alto, CA, USA) according to instructions provided by the manufacturer. The fragment size and concentration of separated samples was analyzed using Agilent ProSize data analysis software (version 3.0, Agilent Technologies, Palo Alto, CA, USA) and the data displayed as an electropherogram. Smear analysis was used to determine DNA concentration within set size ranges of 40–450 bp (small DNA fragments) and 1000–60,000 bp (large DNA fragments). These concentrations were then expressed as percentages of the total DNA concentration to estimate the contribution of small and large DNA fragments to the overall DNA content of the sample. The ProSize software was also used to generate overlays of electropherograms for comparison of DNA profiles between samples.

### 4.7. Droplet Digital PCR

Droplet digital PCR (ddPCR) was performed using the QX200 Droplet Generator and QX200 Droplet Reader from Bio-Rad. The amount of amplifiable DNA extracted from plasma was quantified using the Taqman™ Copy Number RNase P Detection kit (cat# 4403326, ThermoFisher Scientific Waltham, MA, USA) with comparison to a standard curve prepared from human genomic DNA to extrapolate sample DNA concentration. DNA mutations detected by NGS were confirmed by ddPCR using Taqman™ mutation assays, including *PIK3CA p.E545K* (cat# Hs000000086_rm, ThermoFisher), *EGFR p.L858R* (cat # dHsaCP2000021 and dHsaCP2000022, Bio-Rad), and *TP53 p.R248Q* (cat# dHsaCP2000127 and dHsaCP2000128, Bio-Rad). *KRAS* mutations were detected by ddPCR using primers and probes designed and purchased from Integrated DNA Technologies (Coralville, IA, USA) and included: Forward Primer: 5ʹ-AACCTTATGTGTGACATGTTCT-3ʹ; Reverse Primer: 5ʹ-GATTCTGAATTAGCTGTATCGTCAAG-3ʹ; locked nucleic acid (LNA) probes (where LNA is indicated by +) for *KRAS p.G12V* 5ʹ-FAM/AG+CT+G+T+TGG+CG/IABkFQ-3ʹ; *KRAS p.G12C* 5ʹ-FAM/AG+C+T+T+GT+G+GC/IABkFQ-3ʹ; and *KRAS* wt probe 5ʹ-HEX/AGCT+G+G+TG+GC/IABkFQ-3ʹ.

### 4.8. Next-Generation Sequencing

Up to 50 ng of total nucleic acids extracted from plasma was used for NGS library preparation based on the Oncomine™ Lung Total Nucleic Acid assay (ThermoFisher Scientific). RNA was first reverse transcribed using Superscript Vilo master mix and then target amplification of the resulting DNA/cf-DNA mixture was performed using the Oncomine™ Lung Cell-Free Total Nucleic Acid Panel (ThermoFisher Scientific). Amplicons were then purified using Ampure Xp beads (Beckman Coulter, Brea, CA, USA,) and further amplified using tag sequencing barcode adapted primer (ThermoFisher Scientific). Libraries were then purified and size selected by using Ampure XP beads. The library quality was assessed using Ion Library Taqman™ Quantification Kit (ThermoFisher Scientific) and individual libraries were diluted, pooled and normalized to 50 pM for downstream template preparation. Templating was done on Ion Chef Instrument using the Ion 510™ & Ion 520™ & Ion 530™ Kit (ThermoFisher Scientific). Sequencing was done on Ion GeneStudio™ S5 system (ThermoFisher Scientific) by multiplexing up to 6 samples using an Ion 530™ chip. Reads were mapped to Oncomine TagSeq Lung v2 DNA Regions v1.0 (5.12, ThermoFisher Scientific, Waltham, MA, USA)) and analyzed using on Ion Torrent suite software 5.10.1 (ThermoFisher Scientific, Waltham, MA, USA). Oncomine Tagseq Lung v2 liquid biopsy plugin from Ion Reporter software (version 1.0, ThermoFisher Scientific, Waltham, MA, USA) was used for variant calling and annotation.

### 4.9. Statistics

All statistical tests were paired t-tests (Wilcoxon matched-pairs ranked test) and were performed in Prism 8 (GraphPad, San Diego, CA, USA). The sensitivity and specificity of *EGFR* mutation detection in peptide-affinity liquid biopsy sam)ples was determined using the chi-square test with tissue biopsy as the comparator. True positives were considered to be those mutations identified in both tissue and liquid biopsy while true negatives were considered to be those samples in which a mutation was not identified in either tissue or liquid biopsy. The concordance rate between liquid and tissue biopsies was defined as the ratio resulting from the sum of true positive and true negative to total number of genetic alterations included in the analysis. The correlation of NGS sequencing parameters was perform with Pearson Correlation test using R (version 4.0, 2020) (R Core Team, under general public license).

## Figures and Tables

**Table 1:** Clinicopathological Characteristics of NSCLC Patients.

NSCLC Patient Characteristics (*n* = 20)		Overall
Age in years	Mean	65.6
	Median	67
	Range	37–86
Gender	Male (%)	12/20 (60%)
	Female (%)	8/20 (40%)
Cancer Type	NSCLC	20/20 (100%)
	Adenocarcinoma (%)	18/20 (90%)
	Adenosquamous (%)	1/20 (5%)
	Sarcamatoid (%)	1/20 (5%)
Disease Stage	Stage III	4/20 (20%)
	Stage IV	16/20 (80%)
Treatment History	Newly Diagnosed	16/20 (80%)
	Enrolled After Recurrence	4/20 (20%)
Smoking	History of Smoking	17/20 (85%)
	Non-Smoker	2/20 (10%)
	Unknown	1/20 (5%)

**Table 2:** Molecular Profiles of NSCLC Patients.

Patient Molecular Profiles	Overall
No mutation or fusion	4/20 (20%)
*kRAS* mutation	7/20 (35%)
*PIK3CA* mutation	4/20 (20%)
*TP53* mutation	6/20 (30%)
*EGFR* mutation or deletion	6/20 (30%)

**Table 3:** Comparison of *EGFR* Detection in Tissue Biopsy and PA.

Patient n	Mutation Observed	Tissue (MAF)	PA-TNA (MAF)
1	*EGFR*-L858R	22 %	0.7 %
2	*EGFR*-L858R	30 %	Not detected
3	*EGFR*-E746A_A750del	25.8 %	8.2 %
4	*EGFR*-E746A_A750del	65 %	7.3 %
5	*EGFR*-L858R	78.8 %	16.8 %
6	*EGFR*-L858R	24.7 %	0.5 %

**Table 4:** Sensitivity and Specificity of *EGFR* Detection Using PA.

EGFR Detection NSCLC Patients Using PA	
Sensitivity	83.3% (5/6)
Specificity	100% (14/14)

## Supplementary Materials

Supplementary Materials can be found at https://www.mdpi.com/1422-0067/21/23/9083/s1.

## Abbreviations

NSCLC	Non-Small Cell Lung Cancer
cf-DNA	cell-free DNA
EV	Extracellular vesicle
PA	Peptide-affinity
SNV	Single nucleotide variant
TNA	Total nucleic acid
cf-TNA	Cell-free total nucleic acid
PA-TNA	Peptide-affinity total nucleic acid
*EGFR*	Epidermal Growth Factor Receptor
NGS	Next-generation sequencing
*ALK*	Anaplastic lymphoma kinase
ctDNA	Circulating tumour DNA
S-EVs	Small extracellular vesicles
L-EVs	Large extracellular vesicles
HS	High sensitivity
PCR	Polymerase chain reaction
ddPCR	Droplet digital PCR
MAF	Mutant allele frequency
MW	Molecular weight
DNA	Deoxyribonucleic acid
RNA	Ribonucleic acid
CNV	Copy number variant
EDTA	Ethylenediaminetetracetic acid
WBC	White blood cell
miRNA	microRNA

## References

[1] Bray F., Ferlay J., Soerjomataram I., Siegel R.L., Torre L.A., Jemal A. (2018). Global Cancer Statistics 2018: GLOBOCAN Estimates of Incidence and Mortality Worldwide for 36 Cancers in 185 Countries. CA Cancer J. Clin..

[2] Jemal A., Bray F., Center M.M., Ferlay J., Ward E., Forman D. (2011). Global Cancer Statistics. CA Cancer J. Clin..

[3] Halliday P.R., Blakely C.M., Bivona T.G. (2019). Emerging Targeted Therapies for the Treatment of Non-Small Cell Lung Cancer. Curr. Oncol. Rep..

[4] Valentino F., Borra G., Allione P., Rossi L. (2018). Emerging Targets in Advanced Non-Small-Cell Lung Cancer. Future Oncol..

[5] Sholl L.M., Aisner D.L., Allen T.C., Beasley M.B., Borczuk A.C., Cagle P.T., Capelozzi V., Dacic S., Hariri L., Kerr K.M. (2016). Programmed Death Ligand-1 Immunohistochemistry—A New Challenge for Pathologists: A Perspective From Members of the Pulmonary Pathology Society. Arch. Pathol. Lab. Med..

[6] Mathai R., Vidya R., Reddy B., Thomas L., Udupa K., Kolesar J., Rao M. (2019). Potential Utility of Liquid Biopsy as a Diagnostic and Prognostic Tool for the Assessment of Solid Tumors: Implications in the Precision Oncology. J. Clin Med..

[7] Cescon D.W., Bratman S.V., Chan S.M., Siu L.L. (2020). Circulating Tumor DNA and Liquid Biopsy in Oncology. Nat. Cancer.

[8] Weber B., Meldgaard P., Hager H., Wu L., Wei W., Tsai J., Khalil A., Nexo E., Sorensen B.S. (2014). Detection of EGFR Mutations in Plasma and Biopsies from Non-Small Cell Lung Cancer Patients by Allele-Specific PCR Assays. BMC Cancer.

[9] Leighl N.B., Page R.D., Raymond V.M., Daniel D.B., Divers S.G., Reckamp K.L., Villalona-Calero M.A., Dix D., Odegaard J.I., Lanman R.B. (2019). Clinical Utility of Comprehensive Cell-Free DNA Analysis to Identify Genomic Biomarkers in Patients with Newly Diagnosed Metastatic Non–Small Cell Lung Cancer. Clin. Cancer Res..

[10] Diehl F., Schmidt K., Choti M.A., Romans K., Goodman S., Li M., Thornton K., Agrawal N., Sokoll L., Szabo S.A. (2008). Circulating Mutant DNA to Assess Tumor Dynamics. Nat. Med..

[11] Volik S., Alcaide M., Morin R.D., Collins C. (2016). Cell-Free DNA (CfDNA): Clinical Significance and Utility in Cancer Shaped By Emerging Technologies. Mol. Cancer Res..

[12] Thompson J.C., Yee S.S., Troxel A.B., Savitch S.L., Fan R., Balli D., Liberman D.B., Morrissette J.D., Evans T.L., Bauml J. (2016). Detection of Therapeutically Targetable Driver and Resistance Mutations in Lung Cancer Patients by Next-Generation Sequencing of Cell-Free Circulating Tumor DNA. Clin. Cancer Res..

[13] Castro-Giner F., Gkountela S., Donato C., Alborelli I., Quagliata L., Ng C., Piscuoglio S., Aceto N. (2018). Cancer Diagnosis Using a Liquid Biopsy: Challenges and Expectations. Diagnostics.

[14] Lim M., Kim C.-J., Sunkara V., Kim M.-H., Cho Y.-K. (2018). Liquid Biopsy in Lung Cancer: Clinical Applications of Circulating Biomarkers (CTCs and CtDNA). Micromachines.

[15] Yoon Y.J., Kim O.Y., Gho Y.S. (2014). Extracellular Vesicles as Emerging Intercellular Communicasomes. BMB Reports.

[16] Théry C., Witwer K.W., Aikawa E., Alcaraz M.J., Anderson J.D., Andriantsitohaina R., Antoniou A., Arab T., Archer F., Atkin-Smith G.K. (2018). Minimal information for studies of extracellular vesicles 2018 (MISEV2018): A position statement of the International Society for Extracellular Vesicles and update of the MISEV2014 guidelines. J. Extracell. Vesicles.

[17] Voloshin T., Fremder E., Shaked Y. (2014). Small But Mighty: Microparticles as Mediators of Tumor Progression. Cancer Microenviron..

[18] Ciardiello C., Migliorino R., Leone A., Budillon A. (2020). Large Extracellular Vesicles: Size Matters in Tumor Progression. Cytokine Growth Factor Rev..

[19] Momen-Heravi F., Saha B., Kodys K., Catalano D., Satishchandran A., Szabo G. (2015). Increased Number of Circulating Exosomes and Their MicroRNA Cargos Are Potential Novel Biomarkers in Alcoholic Hepatitis. J. Transl. Med..

[20] Brinton L.T., Sloane H.S., Kester M., Kelly K.A. (2015). Formation and Role of Exosomes in Cancer. Cell. Mol. Life Sci..

[21] Fernando M.R., Jiang C., Krzyzanowski G.D., Ryan W.L. (2017). New Evidence That a Large Proportion of Human Blood Plasma Cell-Free DNA Is Localized in Exosomes. PLoS ONE.

[22] Wang Y., Liu B., Lei H., Zhang B., Huang H., Chen S., Feng Y., Zhu L., Gu Y., Zhang Q. (2018). Nanoscale extracellular vesicle-derived DNA is superior to circulating cell-free DNA for mutation detection in early-stage non-small-cell lung cancer. Ann. Oncol..

[23] Allenson K., Castillo J., Lucas F.A.S., Scelo G., Kim D.U., Bernard V., Davis G., Kumar T., Katz M., Overman M.J. (2017). High prevalence of mutant KRAS in circulating exosome-derived DNA from early-stage pancreatic cancer patients. Ann. Oncol..

[24] Möhrmann L., Huang H.J., Hong D.S., Tsimberidou A.M., Funda M.-B., Piha-Paul S.A., Subbiah V., Karp D.D., Naing A., Krug A. (2018). Liquid Biopsies Using Plasma Exosomal Nucleic Acids and Plasma Cell-Free DNA Compared with Clinical Outcomes of Patients with Advanced Cancers. Clin. Cancer Res..

[25] Lee J.S., Hur J.Y., Kim I.A., Kim H.J., Choi C.M., Lee J.C., Kim W.S., Lee K.Y. (2018). Liquid Biopsy Using the Supernatant of a Pleural Effusion for EGFR Genotyping in Pulmonary Adenocarcinoma Patients: A Comparison between Cell-Free DNA and Extracellular Vesicle-Derived DNA. BMC Cancer.

[26] Ghosh A., Davey M., Chute I.C., Griffiths S.G., Lewis S., Chacko S., Barnett D., Crapoulet N., Fournier S., Joy A. (2014). Rapid Isolation of Extracellular Vesicles from Cell Culture and Biological Fluids Using a Synthetic Peptide with Specific Affinity for Heat Shock Proteins. PLoS ONE.

[27] Reddy V.S., Madala S.K., Trinath J., Reddy G.B. (2018). Extracellular Small Heat Shock Proteins: Exosomal Biogenesis and Function. Cell Stress Chaperones.

[28] Foroni C., Zarovni N., Bianciardi L., Bernardi S., Triggiani L., Zocco D., Venturella M., Chiesi A., Valcamonico F., Berruti A. (2020). When Less Is More: Specific Capture and Analysis of Tumor Exosomes in Plasma Increases the Sensitivity of Liquid Biopsy for Comprehensive Detection of Multiple Androgen Receptor Phenotypes in Advanced Prostate Cancer Patients. Biomedicines.

[29] Zocco D., Bernardi S., Novelli M., Astrua C., Fava P., Zarovni N., Carpi F.M., Bianciardi L., Malavenda O., Quaglino P. (2020). Isolation of Extracellular Vesicles Improves the Detection of Mutant DNA from Plasma of Metastatic Melanoma Patients. Sci. Rep..

[30] Roy J., Taylor C., Beauregard A.P., Dhadi S.R., Ayre D.C., Fry S., Chacko S., Wajnberg G., Joy A., Mai-Thi N.N. A multiparametric extraction method for the molecular characterization of Vn96-isolated plasma extracellular vesicles and cell free DNA that enables multi-omic profiling. Sci. Rep..

[31] Vagner T., Spinelli C., Minciacchi V.R., Balaj L., Zandian M., Conley A., Zijlstra A., Freeman M.R., Demichelis F., De S. (2018). Large Extracellular Vesicles Carry Most of the Tumour DNA Circulating in Prostate Cancer Patient Plasma. J. Extracell. Vesicles.

[32] Doyle L., Wang M. (2019). Overview of Extracellular Vesicles, Their Origin, Composition, Purpose, and Methods for Exosome Isolation and Analysis. Cells.

[33] Sorber L., Zwaenepoel K., Jacobs J., De Winne K., Goethals S., Reclusa P., Van Casteren K., Augustus E., Lardon F., Roeyen G. (2019). Circulating Cell-Free DNA and RNA Analysis as Liquid Biopsy: Optimal Centrifugation Protocol. Cancers.

[34] Han D.S.C., Ni M., Chan R.W.Y., Chan V.W.H., Lui K.O., Chiu R.W.K., Lo Y.M.D. (2020). The Biology of Cell-Free DNA Fragmentation and the Roles of DNASE1, DNASE1L3, and DFFB. Am. J. Hum. Genet..

[35] Gutierrez M.E., Choi K., Lanman R.B., Licitra E.J., Skrzypczak S.M., Pe Benito R., Wu T., Arunajadai S., Kaur S., Harper H. (2017). Genomic Profiling of Advanced Non–Small Cell Lung Cancer in Community Settings: Gaps and Opportunities. Clin. Lung Cancer.

[36] Thakur B.K., Zhang H., Becker A., Matei I., Huang Y., Costa-Silva B., Zheng Y., Hoshino A., Brazier H., Xiang J. (2014). Double-stranded DNA in exosomes: A novel biomarker in cancer detection. Cell Res..

[37] Warton K., Yuwono N.L., Cowley M.J., McCabe M.J., So A., Ford C.E. (2017). Evaluation of Streck BCT and PAXgene Stabilised Blood Collection Tubes for Cell-Free Circulating DNA Studies in Plasma. Mol. Diagn. Ther..

[38] Witwer K.W., Buzás E.I., Bemis L.T., Bora A., Lässer C., Lötvall J., Hoen E.N.N., Piper M.G., Sivaraman S., Skog J. (2013). Standardization of sample collection, isolation and analysis methods in extracellular vesicle research. J. Extracell. Vesicles.

[39] Risberg B., Tsui D.W.Y., Biggs H., De Almagro A.R.-V.M., Dawson S.-J., Hodgkin C., Jones L., Parkinson C., Piskorz A., Marass F. (2018). Effects of Collection and Processing Procedures on Plasma Circulating Cell-Free DNA from Cancer Patients. J. Mol. Diagn..

[40] Askeland A., Borup A., Østergaard O., Olsen J.V., Lund S.M., Christiansen G., Kristensen S.R., Heegaard N.H.H., Pedersen S. (2020). Mass-Spectrometry Based Proteome Comparison of Extracellular Vesicle Isolation Methods: Comparison of ME-Kit, Size-Exclusion Chromatography, and High-Speed Centrifugation. Biomedicines.

[41] Zhou B., Xu K., Zheng X., Chen T., Wang J., Song Y., Shao Y., Zheng S. (2020). Application of Exosomes as Liquid Biopsy in Clinical Diagnosis. Sig. Transduct. Target Ther..

[42] Jeppesen D.K., Fenix A.M., Franklin J.L., Higginbotham J.N., Zhang Q., Zimmerman L.J., Liebler D.C., Ping J., Liu Q., Evans R. (2019). Reassessment of Exosome Composition. Cell.

[43] Yokoi A., Villar-Prados A., Oliphint P.A., Zhang J., Song X., De Hoff P., Morey R., Liu J., Roszik J., Clise-Dwyer K. (2019). Mechanisms of nuclear content loading to exosomes. Sci. Adv..

[44] Hur J.Y., Kim H.J., Lee J.S., Choi C.-M., Lee J.C., Jung M.K., Pack C.G., Lee K.Y. (2018). Extracellular Vesicle-Derived DNA for Performing EGFR Genotyping of NSCLC Patients. Mol. Cancer.

[45] Vasconcelos M.H., Caires H.R., Ābols A., Xavier C.P.R., Linē A. (2019). Extracellular Vesicles as a Novel Source of Biomarkers in Liquid Biopsies for Monitoring Cancer Progression and Drug Resistance. Drug Resist. Updates.

[46] Alborelli I., Generali D., Jermann P., Cappelletti M.R., Ferrero G., Scaggiante B., Bortul M., Zanconati F., Nicolet S., Haegele J. (2019). Cell-free DNA analysis in healthy individuals by next-generation sequencing: A proof of concept and technical validation study. Cell Death Dis..

[47] Zhu G., Ye X., Dong Z., Lu Y.C., Sun Y., Liu Y., McCormack R., Gu Y., Liu X. (2015). Highly Sensitive Droplet Digital PCR Method for Detection of EGFR-Activating Mutations in Plasma Cell–Free DNA from Patients with Advanced Non–Small Cell Lung Cancer. J. Mol. Diagn..

[48] Douillard J.-Y., Ostoros G., Cobo M., Ciuleanu T., Cole R., McWalter G., Walker J., Dearden S., Webster A., Milenkova T. (2014). Gefitinib Treatment in EGFR Mutated Caucasian NSCLC: Circulating-Free Tumor DNA as a Surrogate for Determination of EGFR Status. J. Thorac. Oncol..

[49] Soria-Comes T., Palomar-Abril V., Ureste M.M., Guerola M.T., Maiques I.C.M. (2020). Real-World Data of the Correlation between EGFR Determination by Liquid Biopsy in Non-Squamous Non-Small Cell Lung Cancer (NSCLC) and the EGFR Profile in Tumor Biopsy. Pathol. Oncol. Res..

[50] Luchini C., Veronese N., Nottegar A., Cappelletti V., Daidone M.G., Smith L., Parris C., Brosens L.A.A., Caruso M.G., Cheng L. (2019). Liquid Biopsy as Surrogate for Tissue for Molecular Profiling in Pancreatic Cancer: A Meta-Analysis Towards Precision Medicine. Cancers.

[51] Wyatt A.W., Annala M., Aggarwal R., Beja K., Feng F., Youngren J., Foye A., Lloyd P., Nykter M., Beer T.M. (2017). Concordance of Circulating Tumor DNA and Matched Metastatic Tissue Biopsy in Prostate Cancer. J. Natl. Cancer Inst..

[52] Xia L., Li Z., Zhou B., Tian G., Zeng L., Dai H., Li X., Liu C., Lu S., Xu F. (2017). Statistical Analysis of Mutant Allele Frequency Level of Circulating Cell-Free DNA and Blood Cells in Healthy Individuals. Sci. Rep..

[53] Russano M., Napolitano A., Ribelli G., Iuliani M., Simonetti S., Citarella F., Pantano F., Dell’Aquila E., Anesi C., Silvestris N. (2020). Liquid biopsy and tumor heterogeneity in metastatic solid tumors: The potentiality of blood samples. J. Exp. Clin. Cancer Res..

[54] Que D., Xiao H., Zhao B., Zhang X., Wang Q., Xiao H., Wang G. (2016). EGFR Mutation Status in Plasma and Tumor Tissues in Non-Small Cell Lung Cancer Serves as a Predictor of Response to EGFR-TKI Treatment. Cancer Biol. Ther..

[55] Reclusa P., Taverna S., Pucci M., Durendez E., Calabuig S., Manca P., Serrano M.J., Sober L., Pauwels P., Russo A. (2017). Exosomes as Diagnostic and Predictive Biomarkers in Lung Cancer. J. Thorac. Dis..

[56] Yoshioka Y., Katsuda T., Ochiya T. (2018). Extracellular Vesicles and Encapusulated MiRNAs as Emerging Cancer Biomarkers for Novel Liquid Biopsy. Jpn. J. Clin. Oncol..

[57] Sandfeld-Paulsen B., Aggerholm-Pedersen N., Baek R., Jakobsen K.R., Meldgaard P., Folkersen B.H., Rasmussen T.R., Varming K., Jørgensen M.M., Sorensen B.S. (2016). Exosomal Proteins as Prognostic Biomarkers in Non-Small Cell Lung Cancer. Mol. Oncol..

