# Supplementary figures and images for: Peptide-Affinity Precipitation of Extracellular Vesicles and Cell-Free DNA Improves Sequencing Performance for the Detection of Pathogenic Mutations in Lung Cancer Patient Plasma
Source: Int J Mol Sci. 2020 Nov 29;21(23):9083. doi: 10.3390/ijms21239083 (PMC7730179; doi:10.3390/ijms21239083)

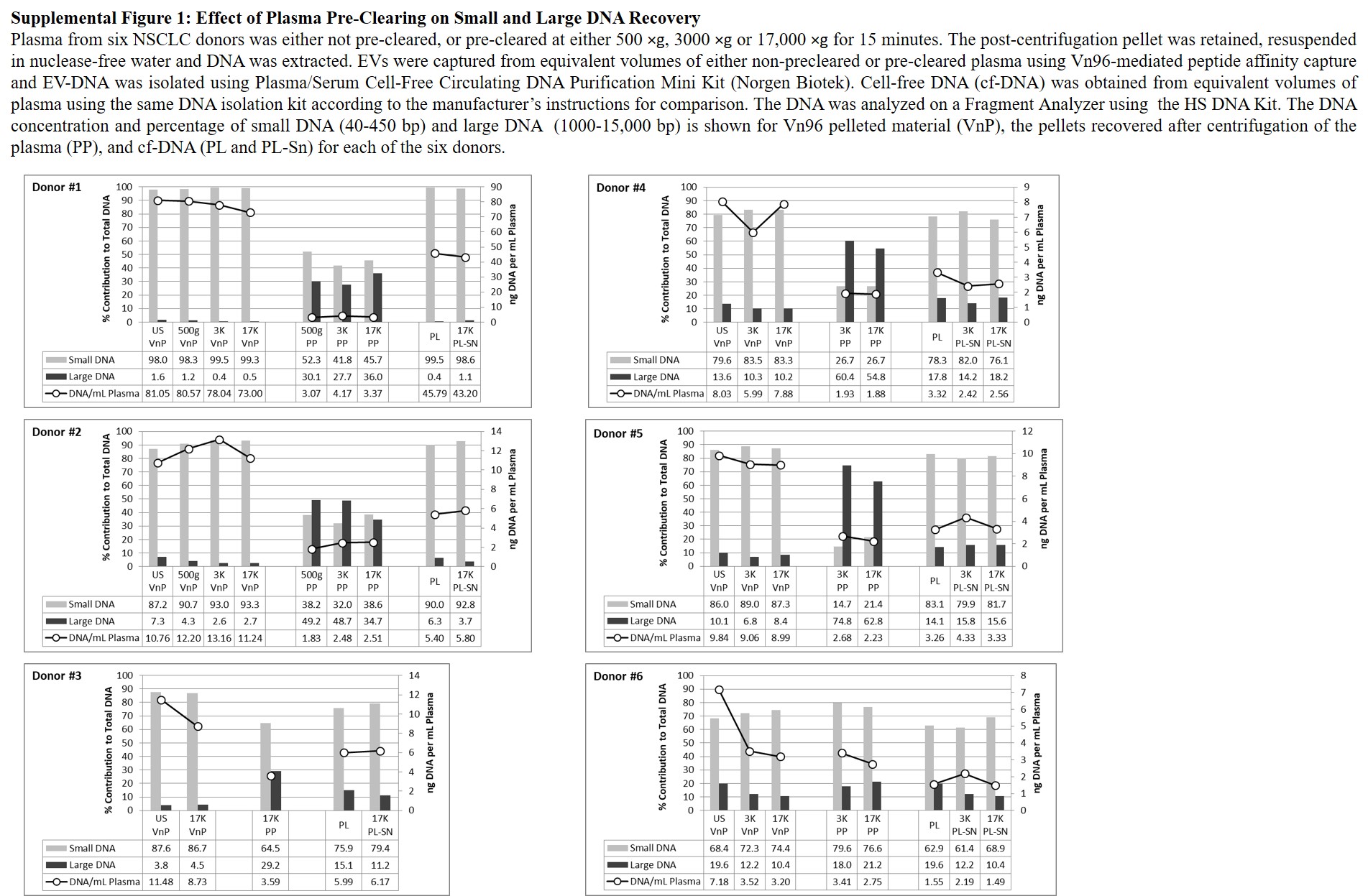

Supplement: Supplementary file 1 [file ijms-21-09083-s001.zip › Sup Fig. 1.jpg]

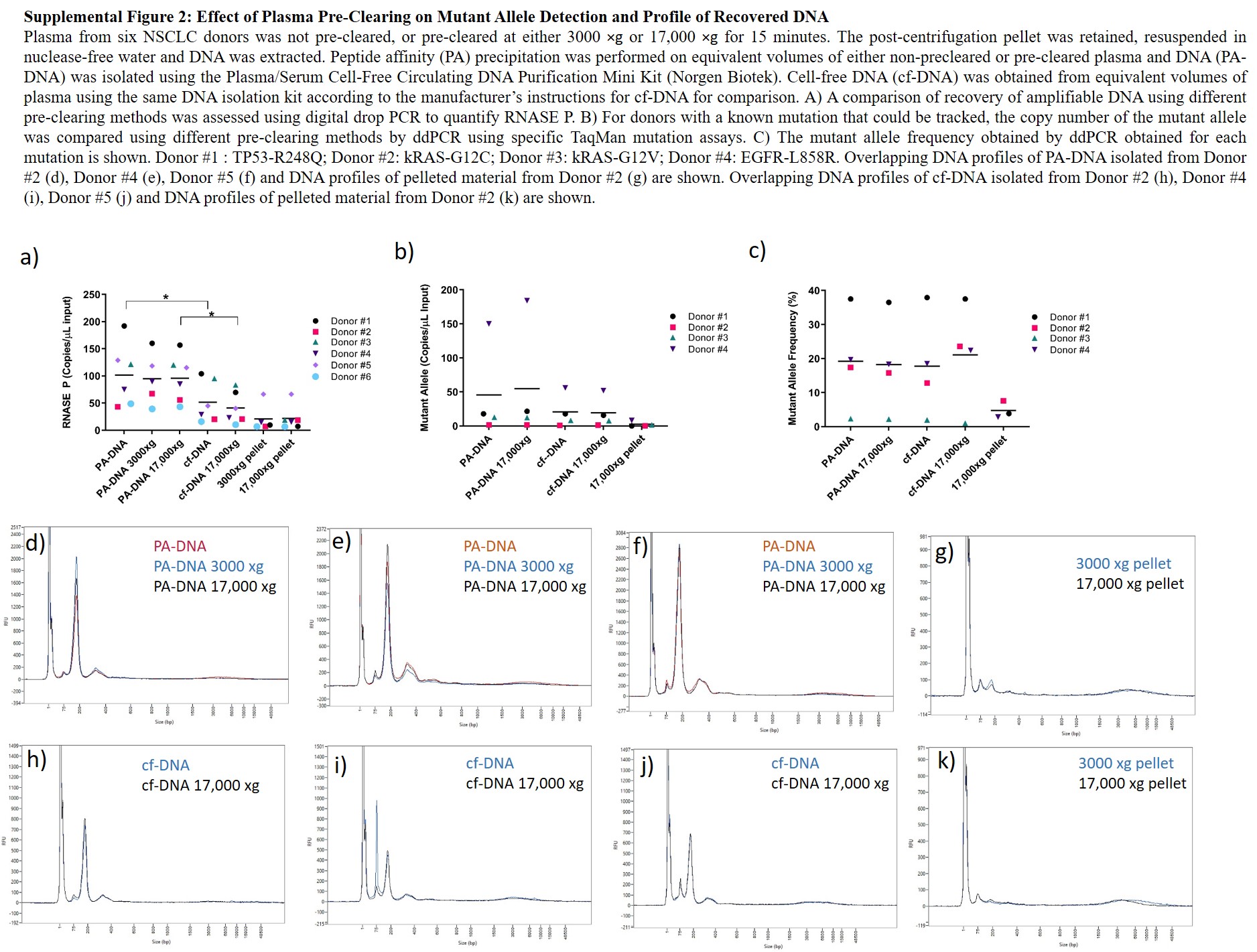

Supplement: Supplementary file 1 [file ijms-21-09083-s001.zip › Sup. Fig. 2.jpg]

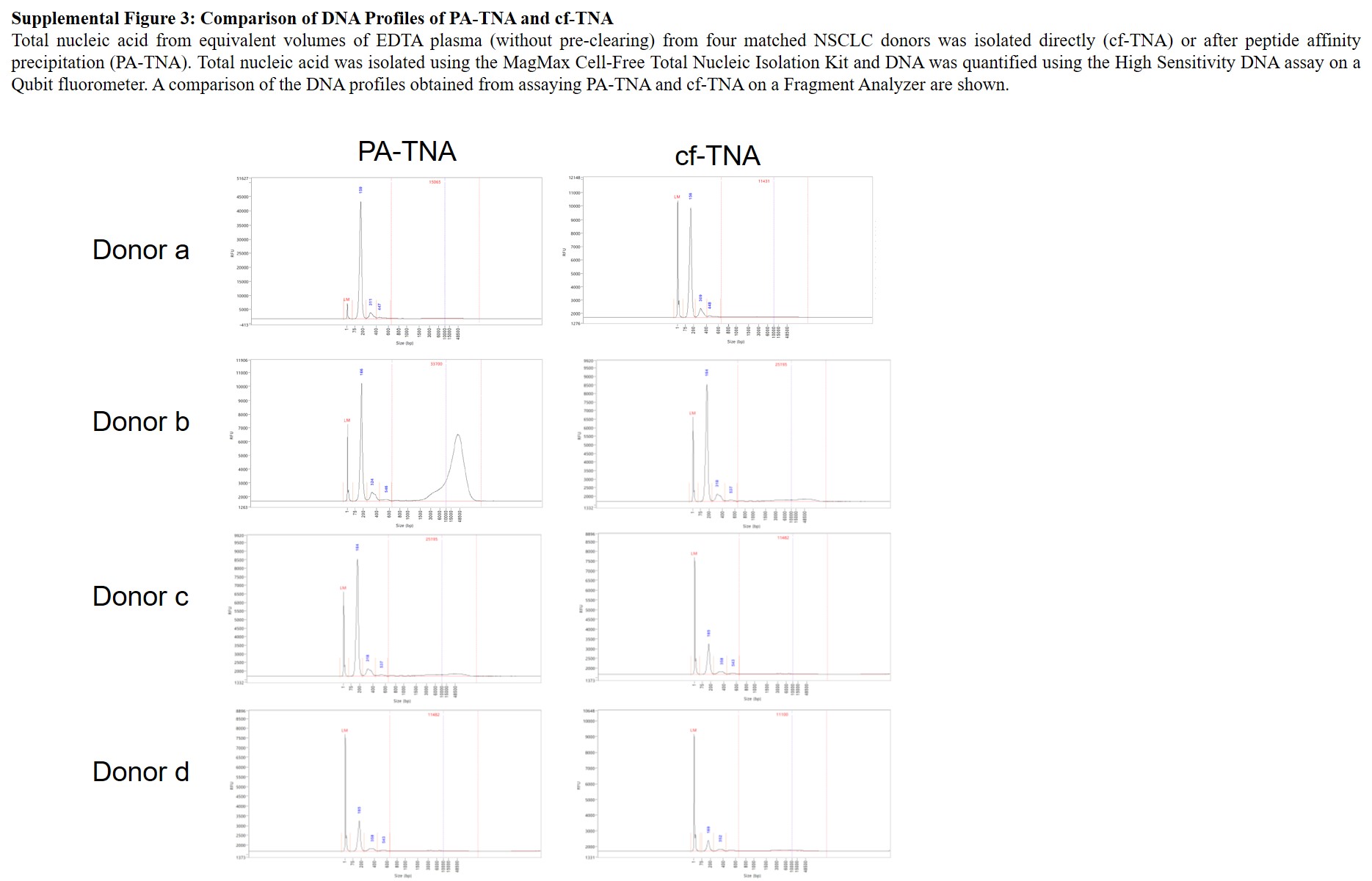

Supplement: Supplementary file 1 [file ijms-21-09083-s001.zip › Sup. Fig. 3.jpg]

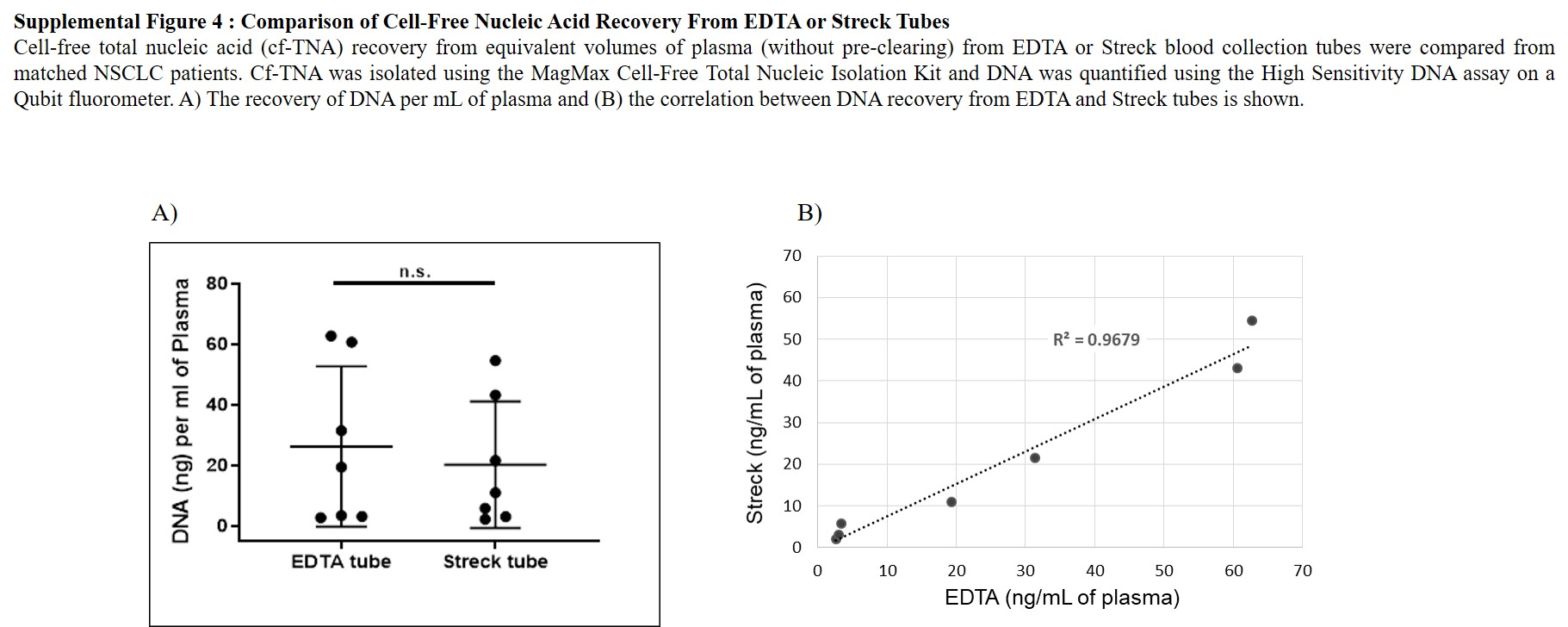

Supplement: Supplementary file 1 [file ijms-21-09083-s001.zip › Sup. Fig. 4.jpg]

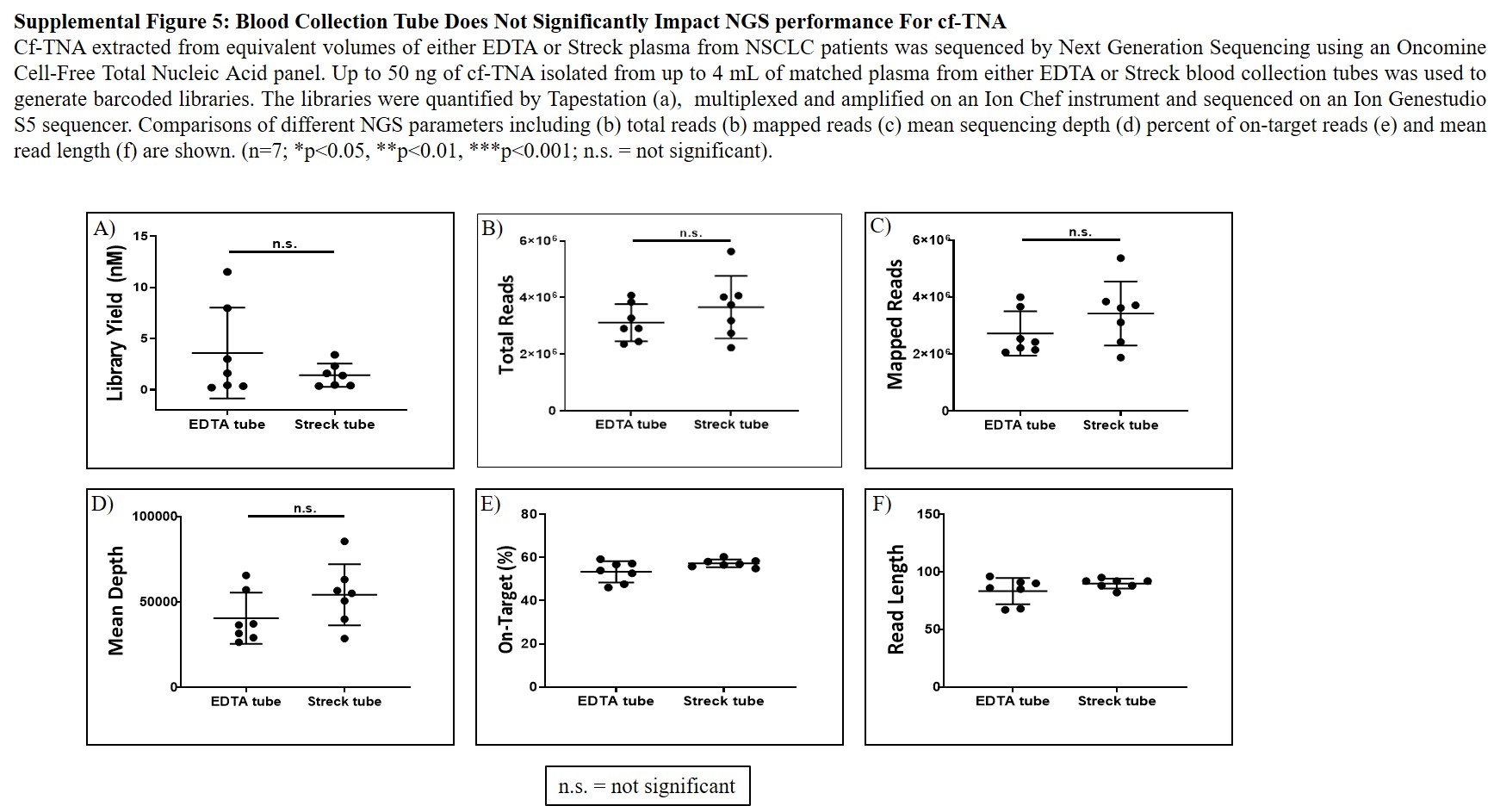

Supplement: Supplementary file 1 [file ijms-21-09083-s001.zip › Sup. Fig. 5.jpg]

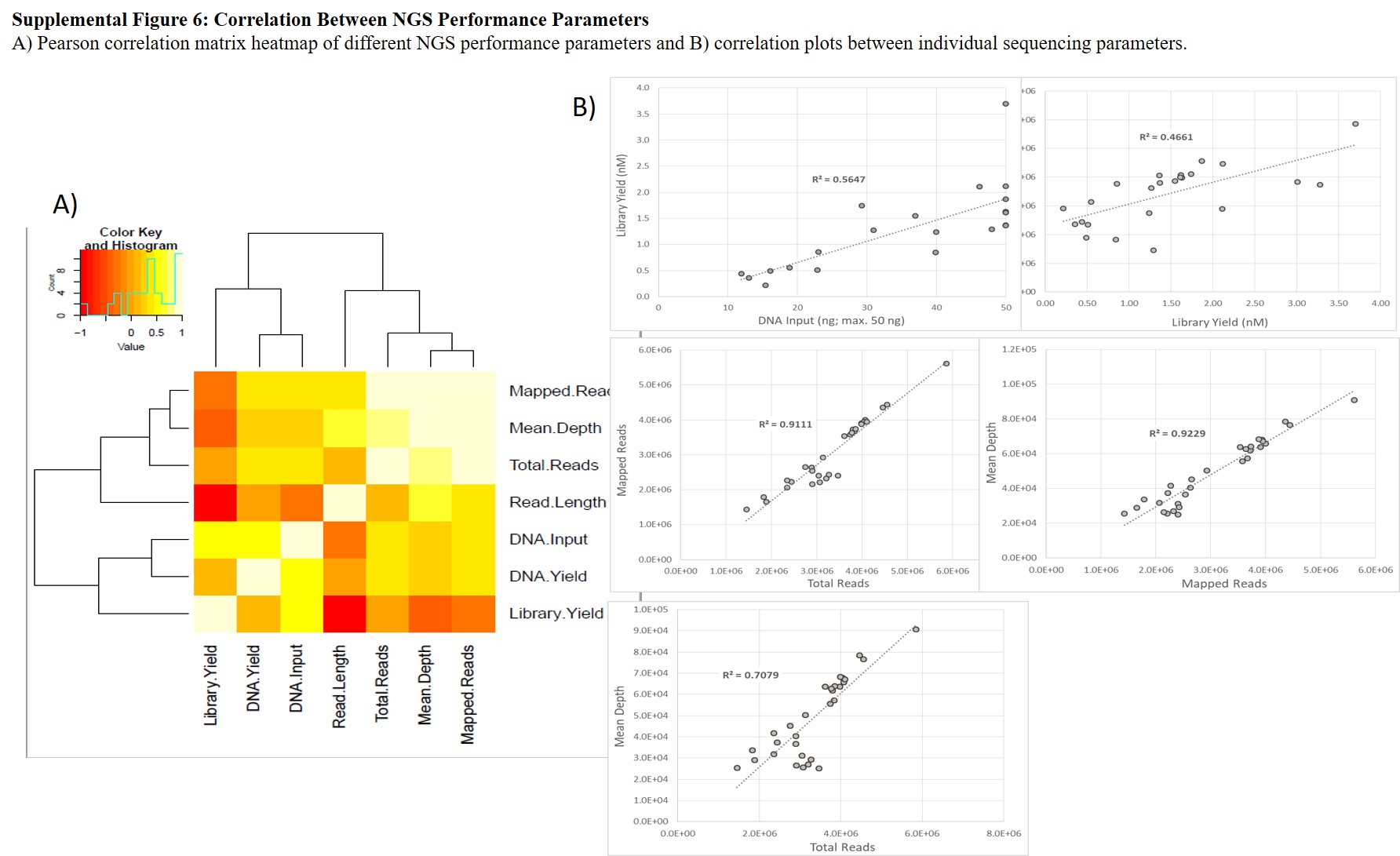

Supplement: Supplementary file 1 [file ijms-21-09083-s001.zip › Sup. Fig. 6 .jpg]

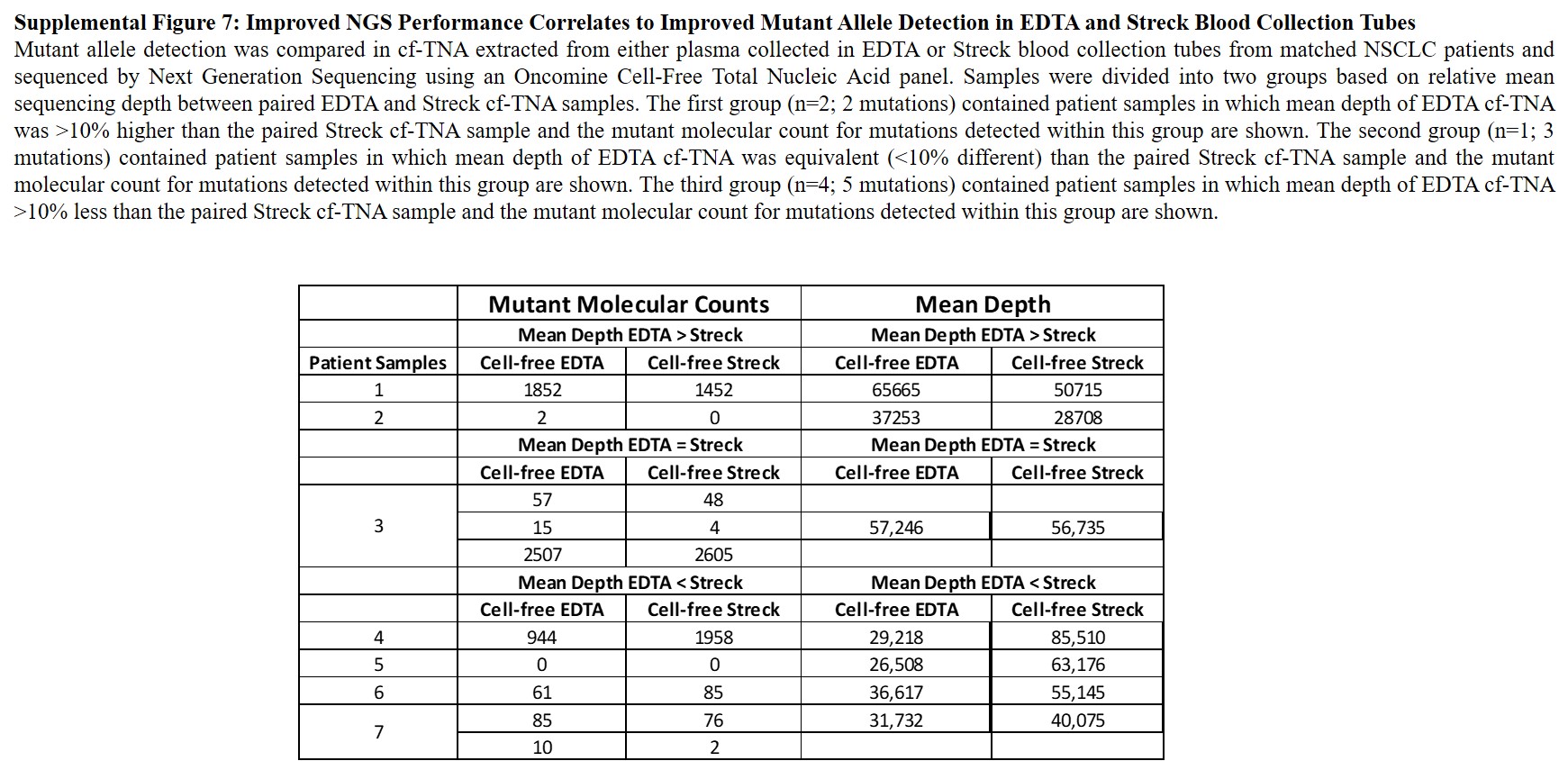

Supplement: Supplementary file 1 [file ijms-21-09083-s001.zip › Sup. Fig. 7.jpg]

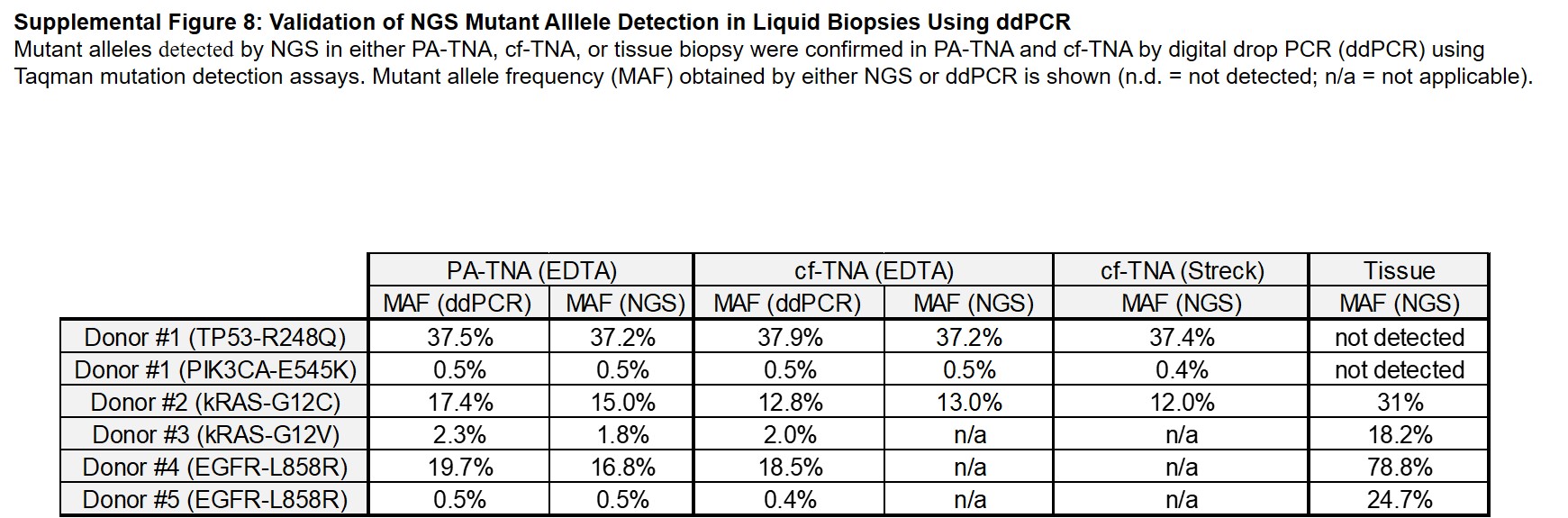

Supplement: Supplementary file 1 [file ijms-21-09083-s001.zip › Sup. Fig. 8.jpg]

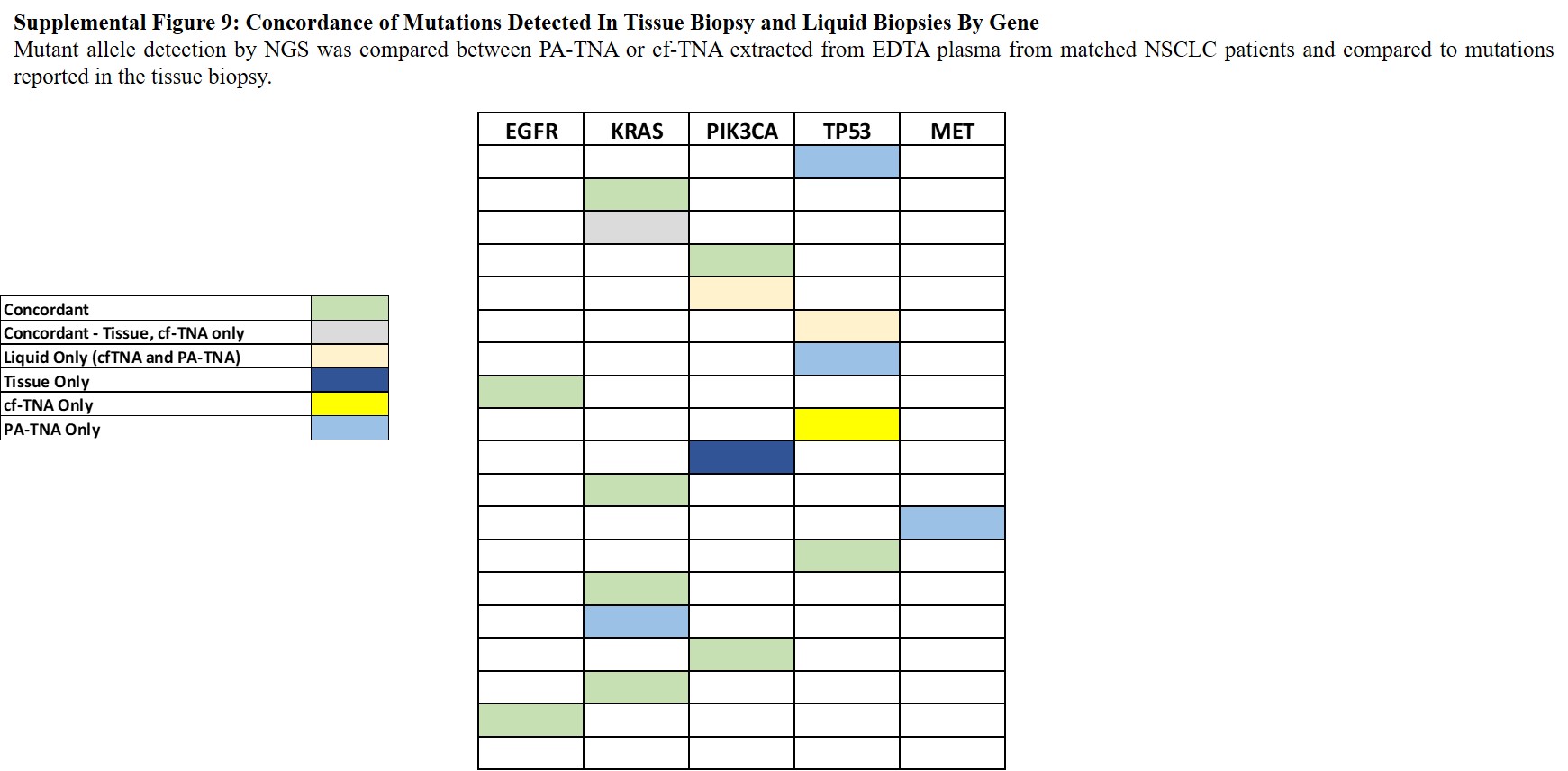

Supplement: Supplementary file 1 [file ijms-21-09083-s001.zip › Sup. Fig. 9.jpg]
